# Increasing protein production by directed vector backbone evolution

**DOI:** 10.1186/2191-0855-3-39

**Published:** 2013-07-26

**Authors:** Felix Jakob, Christian Lehmann, Ronny Martinez, Ulrich Schwaneberg

**Affiliations:** 1 Lehrstuhl für Biotechnologie, RWTH Aachen University, Worringerweg 1, Aachen 52074, Germany; 2 DWI an der RWTH Aachen e.V, Forckenbeckstraße 50, Aachen 52056, Germany

**Keywords:** Directed evolution, epMEGAWHOP, Recombinant protein production, Lipase expression, Cellulase expression, Protease expression

## Abstract

Recombinant protein production in prokaryotic and eukaryotic organisms was a key enabling technology for the rapid development of industrial and molecular biotechnology. However, despite all progress the improvement of protein production is an ongoing challenge and of high importance for cost-effective enzyme production. With the epMEGAWHOP mutagenesis protocol for vector backbone optimization we report a novel directed evolution based approach to increase protein production levels by randomly introducing mutations in the vector backbone. In the current study we validate the epMEGAWHOP mutagenesis protocol for three different expression systems. The latter demonstrated the general applicability of the epMEGAWHOP method. Cellulase and lipase production was doubled in one round of directed evolution by random mutagenesis of pET28a(+) and pET22b(+) vector backbones. Protease production using the vector pHY300PLK was increased ~4-times with an average of ~1.25 mutations per kb vector backbone. The epMEGAWHOP does not require any rational understanding of the expression machinery and can generally be applied to enzymes, expression vectors and related hosts. epMEGAWHOP is therefore from our point of view a robust, rapid and straight forward alternative for increasing protein production in general and for biotechnological applications.

## Introduction

Development of cloning and expression technologies to produce recombinant proteins in prokaryotic and eukaryotic organisms enabled the production of numerous enzymes for diagnostic and industrial applications such as food, leather, textile or detergent industry (Kirk et al. 2002; Rai and Padh 2001). Recombinant production of proteins in prokaryotic hosts is commonly increased by optimization of cultivation or fermentation conditions (temperature, aeration, media composition and/or the fermentation type, such as batch, fed-batch or continuous), codon optimization (Gustafsson et al. 2004; Li et al. 2003), co-expression of chaperones (Thomas et al. 1997), increasing the lifetime of mRNA (Terpe 2006), exchanging/modifying promoters and/or signal peptides (Caspers et al. 2010; Degering et al. 2010; Xue et al. 1997), optimizing the distance between promoter and gene (Brosius et al. 1985), protein fusion technology (e.g. NusA, maltose-binding protein [MBP] or glutathione S-transferase [GST]) (Cabrita et al. 2006), or by metabolic engineering (Heyland et al. 2011; Kabisch et al. 2012).

Directed evolution has become a standard approach to tailor enzyme properties such as activity, solubility, temperature or organic solvent resistance to often non-natural requirements in industrial production. In directed enzyme evolution campaigns enzyme production is often unexpectedly increased in initial rounds (Tee and Schwaneberg 2007). A typical directed evolution experiment comprises three iterative steps of (1) diversity generation, (2) screening to identify improved variants out of a large pool of variants, and (3) isolating the genes encoding for improved variants (Shivange et al. 2009). The screening system plays a pivotal role for reliably identifying improved enzyme variants. Among the screening formats, 96-well microtiter plates are commonly used in directed evolution experiments with throughputs of a few thousand variants per round (Tee and Schwaneberg 2007). Recent developments in high throughput screening technologies comprise throughputs of 10^6^-10^8^ based on flow cytometry (Ruff et al. 2012; Tu et al. 2011) and microfluidics (Fallah-Araghi et al. 2012; Kintses et al. 2012). In this work we report a novel strategy to increase enzyme production in bacterial hosts (*E. coli* and *B. subtilis*), which is based on the ‘megaprimer PCR of whole plasmid method’ (MEGAWHOP) (Miyazaki 2003; Miyazaki 2011). In our modified error-prone MEGAWHOP method (epMEGAWHOP) mutated vector backbone libraries were generated by amplification under error-prone PCR conditions using non-mutated genes as megaprimers, and subsequently screened for increased activity. The general applicability of the developed strategy was proven by using three enzymes (CelA2, BSLA and subtilisin Carlsberg) in three different vector-systems (pET28a(+), pET22b(+) and pHY300PLK) and two different and industrially important expression hosts (*E. coli* (Baneyx 1999; Jana and Deb 2005 and *B. subtilis* (Terpe 2006; Westers et al. 2004)). In all three cases an increased enzyme production was obtained with optimized vector backbones.

## Materials and methods

All chemicals were of analytical-reagent grade or higher quality and were purchased from Carl Roth GmbH (Karlsruhe, Germany), Sigma-Aldrich (Hamburg, Germany) and AppliChem (Darmstadt, Germany). Enzymes were purchased from New England Biolabs (Beverly, USA) and Fermentas (St. Leon-Rot, Germany). Oligonucleotides were purchased from Eurofins MWG Operon (Ebersberg, Germany) in salt-free form. Plasmid extraction and PCR purification kits were purchased from Macherey-Nagel (Düren, Germany). Microtiter plates (Greiner Bio-One GmbH, Frickenhausen, Germany) were incubated in a Multitron II Infors shaker (Infors AG, Bottmingen, Switzerland). DNA concentrations were quantified using a NanoDrop photometer (ND-1000, NanoDrop Technologies, Wilmington, USA). A Mastercycler gradient (Eppendorf, Hamburg, Germany) and thin-wall PCR tubes (Multi ultra-tubes; 0.2 mL; Carl Roth, Germany) were used in all PCRs.

### Strains and plasmids

*E. coli* DH5α, *E. coli* BL21-Gold (DE3) (purchased from Agilent Technologies; Santa Clara, USA) and *B. subtilis* DB104 (Kawamura and Doi 1984) were used in this study as hosts for DNA manipulation and recombinant protein production. For construction of the expression vectors for the *E. coli* strains (DH5α; BL21-Gold (DE3)), and the plasmids pET28a(+) or pET22b(+) (Novagen; Darmstadt, Germany) were used. In case of *B. subtilis* DB104 the shuttle vector pHY300PLK (Takara Bio Inc., Shiga, Japan) was employed. Chemically competent *E. coli* DH5α and *E. coli* BL21-Gold (DE3) cells with determined transformation efficiencies of 3 × 10^7^ and 3 × 10^6^ cfu/μg pUC19, respectively, were prepared in-house using the rubidium chloride method (Hanahan 1983). Transformation of *B. subtilis* DB104 was performed using a recently developed method which is based on natural competence (Vojcic et al. 2012).

### Gene cloning, construction of expression vectors, and sequencing

#### CelA2

The parent *celA2* (GenBank: JF826524.1; (Lehmann et al. 2012)) was ordered as a synthetic gene from GeneArt (Regensburg, Germany) with an optimized codon usage for *E. coli* (GenBank submission number ID1624106) flanked by an *Nco*I and an *Eco*RI restriction site. Additionally, *celA2* contains an N-terminal His-tag, followed by a TEV-protease sequence. After double digestion with *Nco*I and *Eco*RI, the fragment was subcloned into pET28a(+). The generated construct, named pET28a(+)-CelA2, was transformed into *E. coli* DH5α and sequenced to exclude mutations.

#### Bacillus subtilis lipase A (BSLA)

After double digestion of the parent *Bacillus subtilis* lipase A (BSLA) (GenBank: JX048066.1) with *Nde*I and *Xho*I, the fragment was subcloned using T4 DNA ligase into pET22b(+). The resultant recombinant plasmid, named pET22b(+)-BSLA, contains a C-terminal His-tag and a *pelB* leader sequence. The plasmid construct was transformed into *E. coli* DH5α and sequenced to exclude mutations.

#### Subtilisin Carlsberg

After double digestion of a *subtilisin Carlsberg* variant (GenBank: HM147766.1, harboring the silent mutations C479G, T480C, G482A, G869A, T1052C and G1055C) with *Bam*HI and *Xma*I, the gene was subcloned into pHY300PLK using T4 DNA ligase. The resulting recombinant plasmid, named pHYscarlsberg was transformed into *E. coli* DH5α and sequenced to exclude mutations.

DNA sequencing of all three recombinant plasmids was conducted at Eurofins MWG Operon (Ebersberg, Germany) and Clone Manager 9 Professional Edition (Sci-Ed software, Cary, USA) was used for all sequence alignments.

### Generation of error-prone MEGAWHOP libraries

Megaprimers for each target gene were generated by PCR under standard conditions. The amplification of *celA2* and *BSLA* was performed using unmodified DNA primers 5’-GTTATTGCTCAGCGGTGGCAGCAGC-3’ and 5’-TAATACGACTCACTATAGGGGAATTGTGAGCGG-3’ (5 μM each) binding in the T7 promoter and terminator region. The amplification of the *subtilisin Carlsberg* gene includes promoter, pre- and pro-sequence and was performed using unmodified DNA primers 5’-CAGATTTCGTGATGCTTGTCAGG-3’ and 5’-CGTTAAGGGATCAACTTTGGGAG-3’ (5 μM each). For the PCR (98°C for 30 sec, one cycle; 98°C, 15 sec/63°C, 15 sec/68°C, 2 min (*celA2*) or 30 sec (*BSLA, subtilisin Carlsberg*), 25 cycles; 68°C for 10 min, one cycle), PfuS DNA polymerase (2.5 U), dNTP mix (10 mM), template (pET28a(+)-CelA2, pET22b(+)-BSLA and pHYscarlsberg: 30 ng/μL) were used. The PCR products (megaprimers) were purified using a PCR purification kit.

For the epMEGAWHOP PCR (72°C for 5 min, one cycle; 94°C for 90 sec, one cycle; 94°C, 45 sec/60°C, 45 sec/72°C, 5 min, 25 cycles; 72°C for 10 min, one cycle), Taq DNA polymerase (2.5 U), dNTP mix (10 mM) together with plasmid template (pET28a(+)-CelA2, pET22b(+)-BSLA or pHYscarlsberg: 120 ng), megaprimer (pET28a(+)-CelA2, pET22b(+)-BSLA or pHYscarlsberg: 550 ng) and 0.05 mM Mn^2+^ were used. Following the epMEGAWHOP PCR, *Dpn*I digestion (20 U) of the template was performed overnight at 37°C. The epMEGAWHOP PCR product was transformed into *E. coli* DH5α. All colonies from the agar plates were used for plasmid isolation and subsequently transformed into their expression host. The plasmids pET28a(+)-CelA2 and pET22b(+)-BSLA were transformed in *E. coli* BL21-Gold (DE3) and pHYscarlsberg was transformed in *B. subtilis* DB104.

### Indicator plates for pre-screening

In all three screening systems a halo formation can be used to semi-quantify enzymatic activity as indicator for enzyme production.

### Detection of cellulolytic activity

Azo-CarboxyMethyl-Cellulose (Azo-CM-Cellulose, Megazyme, Bray, Ireland) was used as substrate for determining cellulolytic activity (Hughes et al. 2006). LB agar plates supplemented with 0.125% (w/v) Azo-CM-Cellulose, 50 μg/mL kanamycin and 0.1 mM isopropyl-thio-β-D-galactoside (IPTG) were used as indicator plates for pre-screening.

### Detection of lipolytic activity

Tributyrin was used as substrate for lipolytic activity detection (Alquati et al. 2002). LB agar plates supplemented with 100 μg/mL ampicillin, 1.5% (v/v) tributyrin, 0.15% (w/v) gum arabic were used as indicator plates for pre-screening.

### Detection of proteolytic activity

Skim milk was used as substrate for proteolytic activity detection (Sokol et al. 1979). LB agar plates supplemented with 1% (w/v) skim milk and 15 μg/mL tetracycline were used as indicator plates for pre-screening.

### Expression in microtiter plates

#### Growth conditions and expression of CelA2 in 96-well microtiter plates

Colonies displaying cellulolytic activity in the indicator agar plates were transferred into 96-well microtiter plates (flat-bottomed, polystyrene plates). Cultivation and enzyme expression in microtiter plates was performed as previously described (Lehmann et al. 2012) and supernatants of lysates were subsequently used for kinetic characterization.

### Growth conditions and expression of BSLA in 96-well microtiter plates

Colonies displaying lipolytic activity in the indicator agar plates were transferred into 96-well microtiter plates (flat-bottomed, polystyrene plates) containing 200 μL LB medium supplemented with 50 μg/mL ampicillin and incubated (pre-culture; 37°C, 900 rpm, 24 h, 70% humidity). An expression plate contained 150 μL auto-induction medium (1.2% (w/v) casein hydrolysate, 2.4% (w/v) yeast extract, 0.5% (w/v) glycerol, 0.05% (w/v) glucose, 0.02% (w/v) lactose and potassium phosphate buffer (90 mM; pH 7.0) was inoculated with the pre-culture (10 μL) and incubated (main culture; 37°C, 900 rpm, 16 h, 70% humidity). After expression, the microtiter plates were centrifuged (4000 g, 20 min, 4°C) to separate the cells from the culture supernatant. Using the expression vector pET22b(+) including *pelB* sequence, the activity of BSLA can be detected in the culture supernatant (Funke et al. 2003; Khushoo et al. 2004; Yedavalli and Rao 2013). Culture supernatant, including the BSLA was transferred after centrifugation into a 96-well microtiter plate for further analysis.

### Growth conditions and expression of subtilisin Carlsberg in 96-well microtiter plates

Single colonies of *B. subtilis* DB104 which show proteolytic activity on LB skim milk agar plates were transferred into flat-bottom microtiter plates (pre-culture plates) containing buffered LB medium (200 μL; 1% (w/v) tryptone, 0.5% (w/v) yeast extract and 1% (w/v) sodium chloride, 17 mM potassium dihydrogen phosphate, 72 mM dipotassium hydrogen phosphate, 15 μg/mL tetracycline) and incubated (37°C, 900 rpm, 18 h, 70% humidity). The volume of 10 μL pre-culture was used to inoculate the main culture (150 μL buffered LB, 37°C, 900 rpm, 24 h, 70% humidity). After expression, the microtiter plates were centrifuged (4000 g, 20 min, 4°C) to separate cells from secreted protease. The supernatant was transferred into a microtiter plate for further analysis.

### Analysis of the *lacI* repressor system

Performance of the *lacI* repressor system used in the above described growth and assay conditions was analyzed for each pET expression system. CelA2 and BSLA production was performed in TB_kan_- or LB_amp_-medium without supplementing IPTG or the induction components glucose and lactose.

### Screening systems

#### Fluorometric assay for determining cellulolytic activity of CelA2

Cellulolytic activity was measured using 4-Methylumbelliferyl-ß-D-cellobioside (4-MUC) as a fluorogenic substrate (Boschker and Cappenberg 1994; Chernoglazov et al. 1989). The cultivation and enzyme reaction was performed as recently reported (Lehmann et al. 2012).

### Colorimetric assay for determining lipolytic activity of BSLA

The lipolytic activity of BSLA in the supernatant was determined using *p*-nitrophenyl butyrate (*p*NPB) (Shirai et al. 1982). The final reaction contained 0.5 mM *p*-nitrophenyl butyrate dissolved in 10 μL acetonitril, 180 μL triethanolamin buffer (50 mM; pH 7.4) and 10 μL of five times diluted supernatant. The release of *p*-nitrophenolate was continuously monitored at 410 nm in a microtiter plate reader (Tecan Infinite M1000 Pro) for quantifying the BSLA production.

### Colorimetric assay for determining proteolytic activity of subtilisin Carlsberg

Proteolytic activity was determined in a microtiter plate using the synthetic peptide substrate succinyl-L-Ala-L-Ala-L-Pro-L-Phe-*p*-nitroanilide (suc-AAPF-*p*NA) (DelMar et al. 1979). Proteolytic reaction of subtilisin Carlsberg was started by supplementing 10 μL of the 1:20 diluted supernatant to Tris/HCl (100 μL; 100 mM, pH 7.5) containing 1 mM suc-AAPF-*p*NA. The release of *p*-nitroaniline was continuously monitored at 410 nm in a microtiter plate reader (Tecan Infinite M1000 Pro) and the determined activity was used for protease quantification.

### Sodium dodecyl sulfate polyacrylamide gel electrophoresis (SDS-PAGE)

The SDS-PAGE for CelA2 was performed using a stacking gel (5% (w/v) acrylamide) and a separating gel (15% (w/v) acrylamide) (Laemmli 1970). Proteins in the supernatant (including BSLA or subtilisin Carlsberg) were precipitated with trichloroacetic acid (TCA; 30% (v/v); 15 min on ice), resuspended after washing with acetone in Tris/HCl (100 mM; pH 8.0). The proteins in the supernatant after centrifugation of cell lysate (CelA2) as well as the precipitated and resuspended proteins from the cell culture supernatant (BSLA and subtilisin Carlsberg) were loaded and separated by SDS-PAGE. Subsequently, the SDS-PAGE gel was stained with Coomassie brilliant blue.

## Results

In the first sections the development of the epMEGAWHOP protocol is described and the screening for increased activity of the selected cellulase, lipase and protease. Subsequently, the obtained variants showing increased activity were analyzed in detail to validate the epMEGAWHOP method.

### Library generation screening

**Figure 1:**
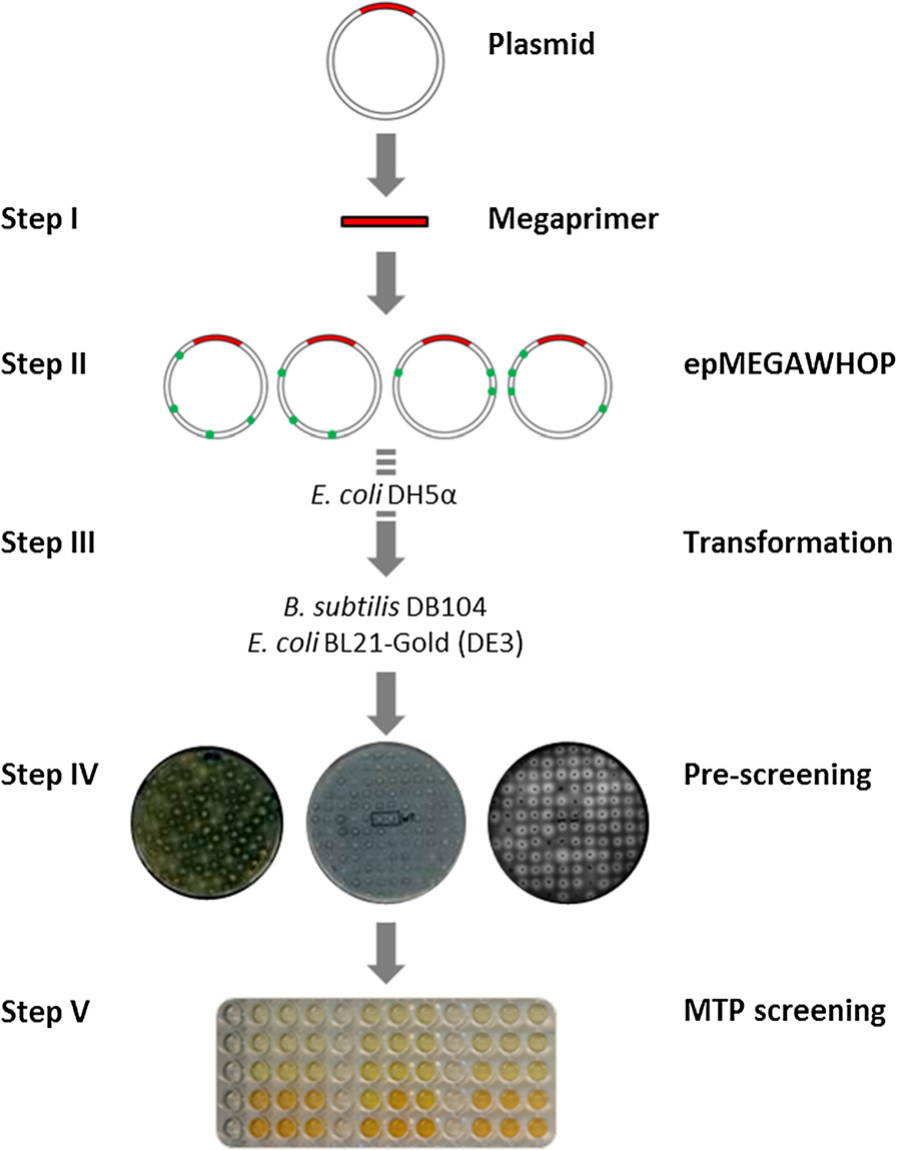
**The five epMEGAWHOP steps to increase protein production by vector backbone mutagenesis. Step I**: megaprimer generation, **Step II**: amplification of the vector backbone under error-prone conditions (0.05 mM Mn^2+^), **Step III**: transformation into *E. coli* DH5α and isolation of plasmids. The expression hosts (*E. coli* BL21-Gold (DE3) or *B. subtilis* DB104) were transformed subsequently with the isolated plasmids, **Step IV**: agar plate pre-screening with selection based on halo formation, and **Step V**: screening in microtiter plate format to quantify increase in enzyme production.

Figure 1 shows the scheme of the developed epMEGAWHOP protocol starting from the generation of megaprimers by PCR (Step I). Followed by an amplification of the whole plasmid under error-prone conditions (0.05 mM Mn^2+^) (Step II) and digestion of the methylated template. In (Step III) the resulting circular DNA is transformed into *E. coli* DH5α cells*.* The plasmids are subsequently isolated from *E. coli* DH5α and transformed into the expression host (*E. coli* BL21-Gold (DE3) or *B. subtilis* DB104) and grown on indicator plates (Step IV). Colonies showing halos are transferred into microtiter plates for quantification of enzymatic activity (Step V). Table 1 summarizes the expression constructs used for epMEGAWHOP development.

**Table 1:** Overview of the expression systems used for developing and validating the epMEGAWHOP method

**Expression host**	**Plasmid**	**Enzyme**	**Location of expressed enzyme**
*E. coli* BL21-Gold (DE3)	pET28a(+)	Cellulase (CelA2)	Intracellular
*E. coli* BL21-Gold (DE3)	pET22b(+)	Lipase A (BSLA)	Periplasmatic
*B. subtilis* DB104	pHY300PLK	Protease (subtilisin Carlsberg)	Extracellular

Approximately 1000 CFU were pre-screened using the corresponding agar plate detection systems for all three expression systems. Ninety promising variants were selected based on halo formation and rescreened more precisely in 96-well microtiter plate format. The most promising candidates (one cellulase, one lipase and one protease) were analyzed in detail to confirm that the increased production can be attributed to an optimized vector backbone. For the latter the wild-type genes (*CelA2, BSLA* and *subtilisin Carlsberg*) were cloned into the mutated backbone as described under Materials and Methods with subsequent rescreening in three 96-well microtiter plate measurements in which each construct was expressed 8 times per plate.

### Activity and expression analysis

**Figure 2:**
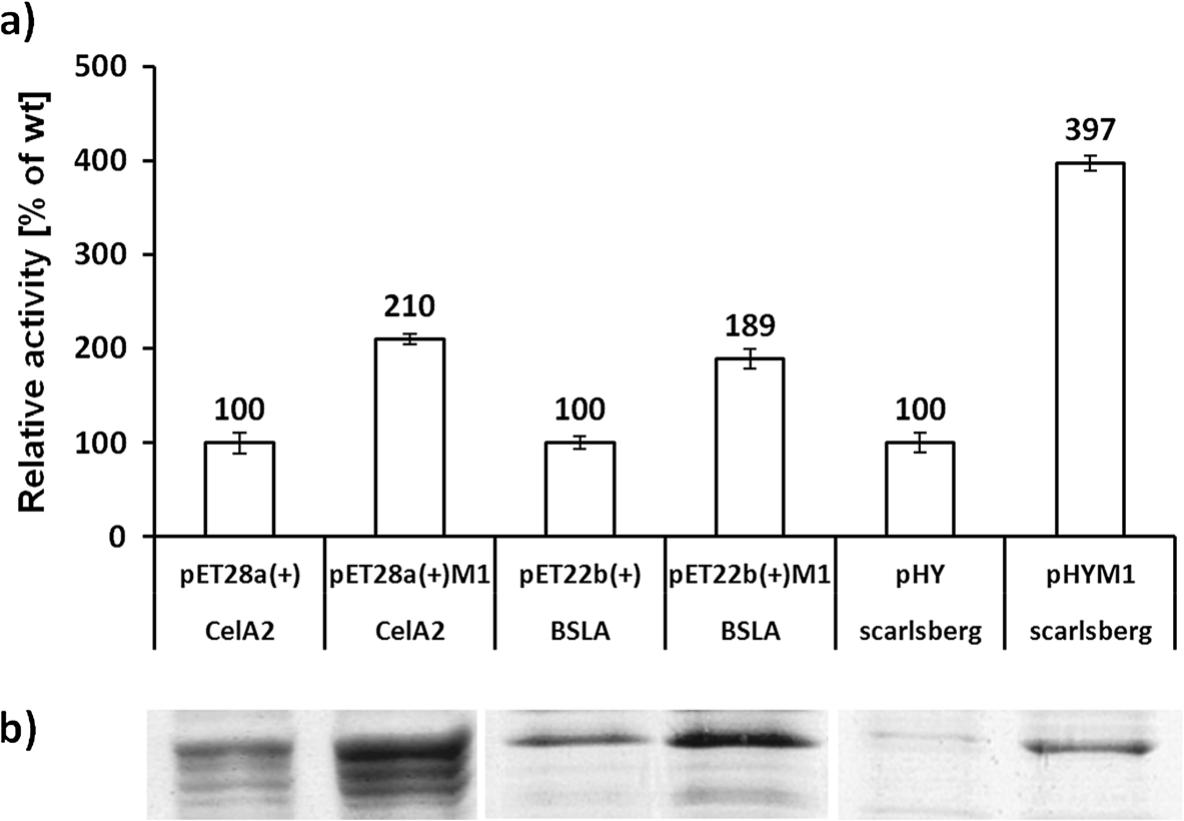
**Activity studies and SDS-PAGE visualization of produced CelA2, BSLA, subtilisin Carlsberg wild type. a)** Activity measurements are performed with not optimized (left column) and mutagenized vector backbones (right column) in 96-well microtiter plate. Constructs with an M1 label habor an epMEGAWHOP optimized vector backbone; **b)** Visualization of production levels of CelA2 (proteins in supernatant of lysate), BSLA and subtilisin Carlsberg (proteins in culture supernatant after precipitation) by SDS-PAGE in combination with Coomassie brilliant blue staining. The reported relative activity values are the average of three 96-well microtiter plate measurements in which each construct was expressed 8 times per plate. Deviations are calculated from the corresponding mean values.

The activity of the three identified variants compared to their parents is shown in Figure 2a. Mutagenized vector backbones of pET28a(+)M1-CelA2 and pET22b(+)M1-BSLA showed compared to the respective ‘wild-type’ a 2-fold increased lipolytic and cellulolytic activity. These activity increases can be correlated to the amount of expressed enzyme which is visualized within a SDS-PAGE in Figure 2b. The mutated vector backbone of pHYM1-scarlsberg showed the highest increase in secreted subtilisin Carlsberg production (4-fold increased proteolytic activity). The increased proteolytic activity correlates well with the increase in the protease content (see SDS-PAGE analysis Figure 2a, b; the whole SDS-PAGE picture is available in the Additional file 1: Figure S1).

### Analysis of the *lacI* repressor system

**Figure 3:**
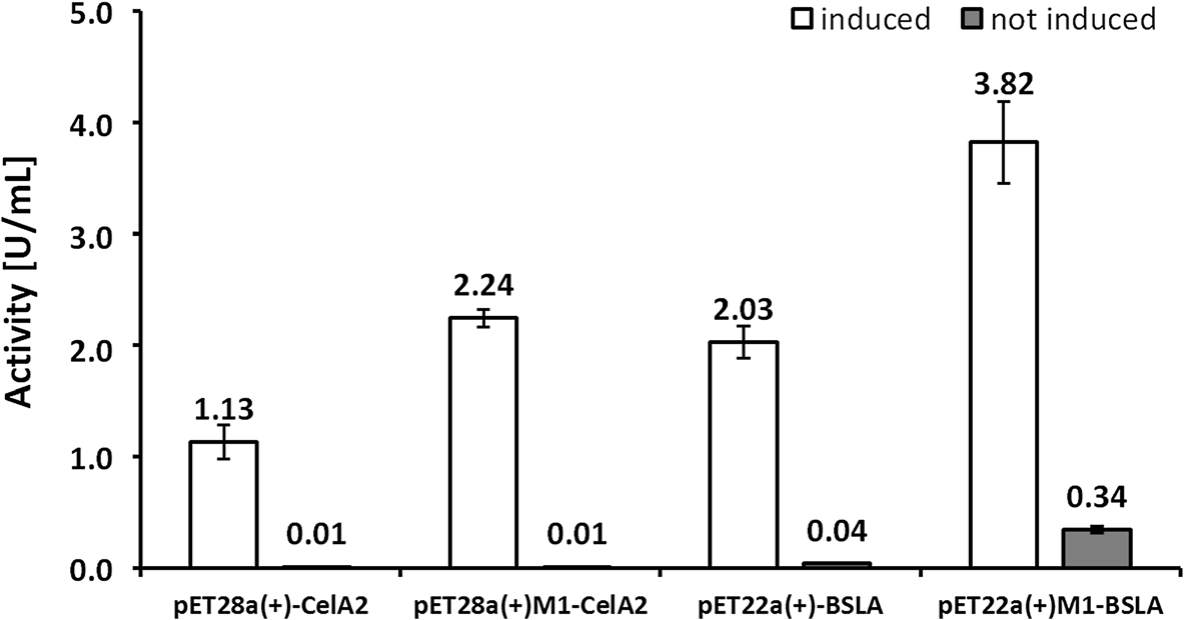
**Functional study of the *lacI *repressor under induced (left bars) and non-induced (right bars) conditions employing pET28a(+)-CelA2, pET28a(+)M1-CelA2, pET22b(+)-BSLA and pET22b(+)M1-BSLA expression systems.** Constructs with an M1 label harbor an epMEGAWHOP optimized vector backbone. Enzyme activity levels were determined with the corresponding screening systems in 96-well microtiter plate formats for CelA2 and BLSA. The reported values are the average of three 96-well microtiter plate measurements in which each hydrolase was expressed 8 times per plate and deviations are calculated from the corresponding mean values.

Expression under inducing and non-inducing conditions was performed to analyze whether the *lacI* repressor contributes to increased production levels. The constructs pET28a(+)-CelA2 and pET22b(+)-BSLA are under the control of *lacI* repressor. Figure 3 shows under non-induced conditions a significant difference in activity/lipase production in the pET22b(+)-BSLA expression vector: the mutated backbone yielded 0.34 U/mL compared to 0.04 U/mL of the ‘parent’ pET22b(+). The latter result proves that *lacI* influences lipase production in pET22b(+)M1-BSLA. Sequencing results confirmed two mutations in *lacI* (see Table 2). An opposite results was found for the pET28a(+)-CelA2 and the pET28a(+)M1-CelA2 expression systems in which under non-induced conditions only very low cellulase activities could be determined (<0.02 U/mL). These results prove that the *lacI* is an effective repressor even in the optimized vector backbone. Sequencing results confirmed that there are no mutations in the *lacI* within the pET28a(+)M1-CelA2 expression system (Table 2). In all four constructs the *lacI* repressor is functional as confirmed by IPTG induction (Figure 3).

**Table 2:** Sequencing results of vector backbones that were subjected to epMEGAWHOP optimization

**pET28a(+)-CelA2**					**pET22b(+)-BSLA**					**pHY-scarlsberg**				
**bp**	**Substitution**			**Region**	**bp**	**Substitution**			**Region**	**bp**	**Substitution**			**Region**
	**wt**	**-**	**M1**			**wt**	**-**	**M1**			**wt**	**-**	**M1**	
488	A	-	T	f1 origin	123	T	-	C	T7 term	209	A	-	G	Tet
850	G	-	C	Kan	2007	A	-	G	na	684	A	-	G	Tet
2233	T	-	C	pBR322 ori	3701	A	-	G	Tet	1584	T	-	C	pTet
2396	T	-	C	na	4563	T	-	C	*lacI*	3238	T	-	C	pAMP
3170	T	-	C	na	4794	C	-	T	*lacI*	4221	T	-	C	na
5193	A	-	T	T7 prom						4332	A	-	-	ori-177

The positions of the mutations in the corresponding vector backbones are based on the sequenced vector backbone prior epMEGAWHOP. Alignments of all three vector systems to the corresponding ‘parents’ are included in Additional file 1: Figure S1. Nucleotide position 1 is the base after the stop codon (ATT, TAA) of the inserted genes.

na: no corresponding function of the gene sequence could be assigned.

Kan/Tet: Kanamycin/Tetracyclin resistance gene.

pTET/pAMP: promoter region Tetracyclin/Ampicilin resistance gene (identified with a promoter prediction tool (Reese 2001)).

### Sequence analysis

Sequence analysis was performed by sequencing the vector backbones and inserts. An average mutation frequency of 1.00-1.25 mutations per 1 kb plasmid DNA was obtained (Additional file 1: Figure S2) and all inserts did not contain a mutation. Table 2 summarizes positions of mutations in the vector backbones and assigns mutations to functional regions. Interestingly, the antibiotic resistance is mutated in all three vector systems, and functional regions such as the origin of replication (in pET28a(+)M1-CelA2; pHYM1-scarlsberg), promoter (pET28a(+)M1-CelA2), repressor (pET22b(+)M1-BSLA), and terminator (pHYM1-scarlsberg).

## Discussion

The MEGAWHOP method is commonly used for cloning random mutagenesis libraries via whole plasmid PCR into the desired expression vector containing the parent gene (Miyazaki 2011). In general enzyme properties such as activity, substrate specificity or stability are improved in directed evolution experiments employing MEGAWHOP (Agudo et al. 2012; Martinez et al. 2013; Sass et al. 2012). The developed epMEGAWHOP does not aim to improve enzymes properties. epMEGAWHOP aims to increase the enzyme production without altering its properties through vector backbone mutagenesis. The error-prone (ep-) label was placed in front of the MEGAWHOP term to point out the main difference that the vector amplification is performed under error-prone conditions.

epMEGAWHOP strategy is a novel strategy to increase enzyme production by optimizing a vector backbone through mutagenesis and high-throughput screening. The epMEGAWHOP method (Figure 1) was validated by employing three different vector constructs with three different enzymes (cellulase: pET28a(+)-CelA2; lipase: pET22b(+)-BSLA; protease: pHY-scarlsberg). A single round of vector backbone mutagenesis and screening yielded significantly increased production levels for all three enzymes (~2-fold for CelA2, ~2-fold for BSLA and ~4-fold for subtilisin Carlsberg). Control experiments through subcloning and sequencing confirmed that only the vector backbones were mutated with an average mutation frequency of ~1.25 mutations per kb. The increase in cellulase, lipase and protease production demonstrate from our point of view that epMEGAWHOP is likely a general method for increasing enzyme production. This is supported by the selection of common expression systems which cover cytosolic (cellulase), periplasmatic (lipase), and extracellular production (protease) in combination with two industrially attractive bacterial hosts (*E. coli* and *B. subtilis*). The main goal of this report is to validate the epMEGAWHOP protocol for vector backbone optimization and not to elucidate in depth the interplay in the complex protein production machinery from the expression to the produced enzyme. The effect of randomly introduced mutations in the vector backbone has to our best knowledge not been investigated in a systematic manner.

Table 2 shows that functional regions such as antibiotic resistance, promoter, repressor or origin of replication were mutated. The mutations can be classified into three groups according to the region in the expression vector: 1) in the kanamycin/tetracycline resistance cassette; 2) once in the promoter and terminator region and 3) twice in the origin of replication. It is known that through the introduction of an expression vector into *E. coli* or other hosts for recombinant protein production, the native cell functions at many levels can be perturbed (e.g. ribosome functions, RNA turnover as well as energy and intermediary metabolism of the cell) (Bailey 1993; Hoffmann et al. 2002; Lin-Chao et al. 2006). This effect is very difficult, however, to predict or attribute to a specific change in the host metabolism, due to the complexity of the system. For example, a reduced but sufficient expression of the resistant marker could allow spending more metabolic resources into expressing the recombinant enzyme. On the other hand, a higher resistance marker expression could allow a higher specific growth rate of the cells which directly correlates with the rate of recombinant protein synthesis (Hoffmann and Rinas 2004).

Elucidating the influence of each mutation and each region on the cellulase, lipase, and protease production would include the introduction of the individual mutations in the parental vector backbones to gain a deeper understanding on the complex machinery from expression system to produced enzyme.

A main prerequisite for a successful epMEGAWHOP experiments is a reliable high-throughput screening system which allows an identification of improved expression variants. It is likely that the mutational load has to be optimized depending on the vector size; an average mutation frequency of 1.25 mutations per 1 kb prove to be efficient to increase enzyme production and all three sequenced vector backbones contained five to six mutations.

In essence, we developed a novel method to increase recombinant protein production based on the random mutagenesis of vector backbones (epMEGAWHOP). The general applicability of epMEGAWHOP was validated by increasing the protein production after one round of directed evolution for *E. coli* BL21-Gold (DE3)/pET28a(+)-CelA2, *E. coli* BL21-Gold (DE3)/pET22b(+)-BSLA and *B. subtilis* DB104/pHY-scarlsberg. The main advantage of epMEGAWHOP is that it doesn’t require a rational understanding of the expression machinery and can generally be applied to enzymes, expression vectors and related hosts. Changing expression vector or promoter systems to achieve higher enzyme yields often requires re-optimization of media composition, induction times and cell densities, together with expression and harvest time, in contrast to epMEGAWHOP. Overall, epMEGAWHOP is a robust, rapid and straight forward alternative and can be used for increasing recombinant protein production.

## Competing interests

The authors declare that they have no competing interests.

## Supplementary Material

Additional file 1: Figure S1 SDS-PAGE of produced cellulase (CelA2), lipase (BSLA) and protease (subtilisin Carlsberg). EV: empty vector control; WT: non-optimized vector constructs; M1: epMEGAWHOP optimized vector constructs. Red arrows indicate the bands of target proteins. Figure S2: Sequencing results: Alignments of all three vector systems compared to the corresponding ‘parents’: pET28a(+)-CelA2 & pET28a(+)M1-CelA2. Click here for file

## References

[B1] Agudo R Roiban GD Reetz MT Achieving Regio- and Enantioselectivity of P450-Catalyzed Oxidative CH Activation of Small Functionalized Molecules by Structure-Guided Directed Evolution ChemBioChem 2012 3 10 1465 1473 10.1002/cbic.201200244 22711296

[B2] Alquati C De Gioia L Santarossa G Alberghina L Fantucci P Lotti M The cold-active lipase of *Pseudomonas fragi*. Heterologous expression, biochemical characterization and molecular modeling Eur J Biochem 2002 3 13 3321 3328 10.1046/j.1432-1033.2002.03012.x 12084074

[B3] Bailey JE Host-vector interactions in *Escherichia coli* Adv Biochem Eng Biotechnol 1993 3 29 52 8460576 10.1007/BFb0007195

[B4] Baneyx F Recombinant protein expression in *Escherichia coli* Curr Opin Biotechnol 1999 3 5 411 421 10.1016/S0958-1669(99)00003-8 10508629

[B5] Boschker HTS Cappenberg TE A Sensitive Method Using 4-Methylumbelliferyl-ß-Cellobiose as a substrate to measure (1,4)-ß-glucanase activity in sediments Appl Environ Microbiol 1994 3 10 3592 3596 16349405 10.1128/aem.60.10.3592-3596.1994 PMC201860

[B6] Brosius J Erfle M Storella J Spacing of the −10 and −35 regions in the tac promoter Effect on its in vivo activity J Biol Chem 1985 3 6 3539 3541 2579077

[B7] Cabrita LD Dai W Bottomley SP A family of *E. coli* expression vectors for laboratory scale and high throughput soluble protein production BMC Biotechnol 2006 3 12 10.1186/1472-6750-6-12 16509985 PMC1420288

[B8] Caspers M Brockmeier U Degering C Eggert T Freudl R Improvement of Sec-dependent secretion of a heterologous model protein in *Bacillus subtilis* by saturation mutagenesis of the N-domain of the AmyE signal peptide Appl Microbiol Biotechnol 2010 3 6 1877 1885 10.1007/s00253-009-2405-x 20077115

[B9] Chernoglazov VM Jafarova AN Klyosov AA Continuous photometric determination of endo-1,4-ß-d-glucanase (cellulase) activity using 4-methylumbelliferyl-ß-d-cellobioside as a substrate Anal Biochem 1989 3 1 186 189 10.1016/0003-2697(89)90222-4 2757193

[B10] Degering C Eggert T Puls M Bongaerts J Evers S Maurer KH Jaeger KE Optimization of protease secretion in *Bacillus subtilis* and *Bacillus licheniformis* by screening of homologous and heterologous signal peptides Appl Environ Microbiol 2010 3 19 6370 6376 10.1128/AEM.01146-10 20709850 PMC2950444

[B11] DelMar EG Largman C Brodrick JW Geokas MC A sensitive new substrate for chymotrypsin Anal Biochem 1979 3 2 316 320 10.1016/S0003-2697(79)80013-5 574722

[B12] Fallah-Araghi A Baret JC Ryckelynck M Griffiths AD A completely in vitro ultrahigh-throughput droplet-based microfluidic screening system for protein engineering and directed evolution Lab Chip 2012 3 5 882 891 10.1039/c2lc21035e 22277990

[B13] Funke SA Eipper A Reetz MT Otto N Thiel W Van Pouderoyen G Dijkstra BW Jaeger KE Eggert T Directed Evolution of an Enantioselective *Bacillus subtilis* Lipase Biocatal Biotransform 2003 3 2 66 73

[B14] Gustafsson C Govindarajan S Minshull J Codon bias and heterologous protein expression Trends Biotechnol 2004 3 7 346 353 10.1016/j.tibtech.2004.04.006 15245907

[B15] Hanahan D Studies on transformation of *Escherichia coli* with plasmids J Mol Biol 1983 3 4 557 580 10.1016/S0022-2836(83)80284-8 6345791

[B16] Heyland J Blank LM Schmid A Quantification of metabolic limitations during recombinant protein production in *Escherichia coli* J Biotechnol 2011 3 2 178 184 10.1016/j.jbiotec.2011.06.016 21723332

[B17] Hoffmann F Rinas U Stress induced by recombinant protein production in *Escherichia coli* Adv Biochem Eng Biotechnol 2004 3 73 92 15217156 10.1007/b93994

[B18] Hoffmann F Weber J Rinas U Metabolic adaptation of *Escherichia coli* during temperature-induced recombinant protein production: 1 Readjustment of metabolic enzyme synthesis Biotechnol Bioeng 2002 3 3 313 319 10.1002/bit.10379 12226864

[B19] Hughes S Riedmuller S Mertens J Li X-L Bischoff K Qureshi N Cotta M Farrelly P High-throughput screening of cellulase F mutants from multiplexed plasmid sets using an automated plate assay on a functional proteomic robotic workcell Proteome Sci 2006 3 10 10.1186/1477-5956-4-10 16670026 PMC1479318

[B20] Jana S Deb JK Strategies for efficient production of heterologous proteins in *Escherichia coli* Appl Microbiol Biotechnol 2005 3 3 289 298 10.1007/s00253-004-1814-0 15635462

[B21] Kabisch J Thurmer A Hubel T Popper L Daniel R Schweder T Characterization and optimization of *Bacillus subtilis* ATCC 6051 as an expression host J Biotechnol 2012 3 97 104 22789474 10.1016/j.jbiotec.2012.06.034

[B22] Kawamura F Doi RH Construction of a *Bacillus subtilis* double mutant deficient in extracellular alkaline and neutral proteases J Bacteriol 1984 3 1 442 444 6434524 10.1128/jb.160.1.442-444.1984 PMC214740

[B23] Khushoo A Pal Y Singh BN Mukherjee KJ Extracellular expression and single step purification of recombinant *Escherichia coli* l-asparaginase II Protein Expr Purif 2004 3 1 29 36 10.1016/j.pep.2004.07.009 15477079

[B24] Kintses B Hein C Mohamed MF Fischlechner M Courtois F Laine C Hollfelder F Picoliter cell lysate assays in microfluidic droplet compartments for directed enzyme evolution Chem Biol 2012 3 8 1001 1009 10.1016/j.chembiol.2012.06.009 22921067

[B25] Kirk O Borchert TV Fuglsang CC Industrial enzyme applications Curr Opin Biotechnol 2002 3 4 345 351 10.1016/S0958-1669(02)00328-2 12323357

[B26] Laemmli UK Cleavage of structural proteins during the assembly of the head of bacteriophage T4 Nature 1970 3 5259 680 685 10.1038/227680a0 5432063

[B27] Lehmann C Sibilla F Maugeri Z Streit WR Domínguez de María P Martinez R Schwaneberg U Reengineering CelA2 cellulase for hydrolysis in aqueous solutions of deep eutectic solvents and concentrated seawater Green Chem 2012 3 10 2719 2726 10.1039/c2gc35790a

[B28] Li A Kato Z Ohnishi H Hashimoto K Matsukuma E Omoya K Yamamoto Y Kondo N Optimized gene synthesis and high expression of human interleukin-18 Protein Expr Purif 2003 3 1 110 118 10.1016/j.pep.2003.08.003 14680947

[B29] Lin-Chao S Chen WT Wong TT High copy number of the pUC plasmid results from a Rom/Rop-suppressible point mutation in RNA II Mol Microbiol 2006 3 22 3385 3393 10.1111/j.1365-2958.1992.tb02206.x 1283002

[B30] Martinez R Jakob F Tu R Siegert P Maurer KH Schwaneberg U Increasing activity and thermal resistance of *Bacillus gibsonii* alkaline protease (BgAP) by directed evolution Biotechnol Bioeng 2013 3 3 711 720 10.1002/bit.24766 23097081

[B31] Miyazaki K Creating random mutagenesis libraries by megaprimer PCR of whole plasmid (MEGAWHOP) Meth Mol Biol 2003 3 23 28 10.1385/1-59259-395-X:23 12824598

[B32] Miyazaki K MEGAWHOP cloning: a method of creating random mutagenesis libraries via megaprimer PCR of whole plasmids Methods Enzymol 2011 3 399 406 21601687 10.1016/B978-0-12-385120-8.00017-6

[B33] Rai M Padh H Expression systems for production of heterologous proteins Curr Sci 2001 3 9 1121 1128

[B34] Ruff AJ Dennig A Wirtz G Blanusa M Schwaneberg U 1. Flow Cytometer-Based High-Throughput Screening System for Accelerated Directed Evolution of P450 Monooxygenases ACS Catalysis 2012 3 12 2724 2728 10.1021/cs300115d

[B35] Sass S Kadow M Geitner K Thompson ML Talmann L Bottcher D Schmidt M Bornscheuer UT A high-throughput assay method to quantify Baeyer-Villiger monooxygenase activity Tetrahedron 2012 3 37 7575 7580 10.1016/j.tet.2012.05.098

[B36] Shirai K Jackson RL Quinn DM Reciprocal effect of apolipoprotein C-II on the lipoprotein lipase-catalyzed hydrolysis of p-nitrophenyl butyrate and trioleoylglycerol J Biol Chem 1982 3 17 10200 10203 7107600

[B37] Shivange AV Marienhagen J Mundhada H Schenk A Schwaneberg U Advances in generating functional diversity for directed protein evolution Curr Opin Chem Biol 2009 3 1 19 25 10.1016/j.cbpa.2009.01.019 19261539

[B38] Sokol PA Ohman DE Iglewski BH A more sensitive plate assay for detection of protease production by *Pseudomanas aeruginosa* J Clin Microbiol 1979 3 4 538 540 110831 10.1128/jcm.9.4.538-540.1979 PMC273070

[B39] Tee KL Schwaneberg U Directed evolution of oxygenases: screening systems, success stories and challenges Comb Chem High Throughput Screen 2007 3 3 197 217 10.2174/138620707780126723 17346119

[B40] Terpe K Overview of bacterial expression systems for heterologous protein production: from molecular and biochemical fundamentals to commercial systems Appl Microbiol Biotechnol 2006 3 2 211 222 10.1007/s00253-006-0465-8 16791589

[B41] Thomas JG Ayling A Baneyx F Molecular chaperones, folding catalysts, and the recovery of active recombinant proteins from *E. coli*. To fold or to refold Appl Biochem Biotechnol 1997 3 3 197 238 10.1007/BF02785589 9276922

[B42] Tu R Martinez R Prodanovic R Klein M Schwaneberg U A flow cytometry-based screening system for directed evolution of proteases J Biomol Screen 2011 3 3 285 294 10.1177/1087057110396361 21335599

[B43] Vojcic L Despotovic D Martinez R Maurer KH Schwaneberg U An efficient transformation method for *Bacillus subtilis* DB104 Appl Microbiol Biotechnol 2012 3 2 487 493 10.1007/s00253-012-3987-2 22395911

[B44] Westers L Westers H Quax WJ *Bacillus subtilis* as cell factory for pharmaceutical proteins: a biotechnological approach to optimize the host organism Biochim Biophys Acta 2004 3 1–3 299 310 15546673 10.1016/j.bbamcr.2004.02.011

[B45] Xue GP Johnson JS Bransgrove KL Gregg K Beard CE Dalrymple BP Gobius KS Aylward JH Improvement of expression and secretion of a fungal xylanase in the rumen bacterium *Butyrivibrio fibrisolvens* OB156 by manipulation of promoter and signal sequences J Biotechnol 1997 3 2 139 148 10.1016/S0168-1656(97)01671-4 9195758

[B46] Yedavalli P Rao NM Engineering the loops in a lipase for stability in DMSO Protein Eng Des Sel 2013 3 4 317 324 10.1093/protein/gzt002 23404771

