# Supplementary material for: Increasing protein production by directed vector backbone evolution
Source: AMB Express. 2013 Jul 26;3:39. doi: 10.1186/2191-0855-3-39 (PMC3750827; doi:10.1186/2191-0855-3-39)
Supplement: Additional file 1: Figure S1 — SDS-PAGE of produced cellulase (CelA2), lipase (BSLA) and protease (subtilisin Carlsberg). EV: empty vector control; WT: non-optimized vector constructs; M1: epMEGAWHOP optimized vector constructs. Red arrows indicate the bands of target proteins. Figure S2: Sequencing results: Alignments of all three vector systems compared to the corresponding ‘parents’: pET28a(+)-CelA2 & pET28a(+)M1-CelA2. [file 2191-0855-3-39-S1.pdf]

Supplementary Material

Increasing protein production by directed vector backbone evolution

Felix Jakob[1,2,A], Christian Lehmann[1, A], Ronny Martinez[1] and Ulrich Schwaneberg[1,2*]

[1]RWTH Aachen University, Worringerweg 1, 52074 Aachen, Germany

[2]DWI an der RWTH Aachen e.V., Forckenbeckstraße 50, 52056 Aachen, Germany

[A]These authors contributed equally to this work

*Corresponding author:

Prof. Dr. Ulrich Schwaneberg

RWTH Aachen University

Lehrstuhl für Biotechnologie

Worringerweg 1

52074 Aachen, Germany

Tel.: +49-241-80 24176

Fax: +49-241-80 22387

E-Mail: u.schwaneberg@biotec.rwth-aachen.de

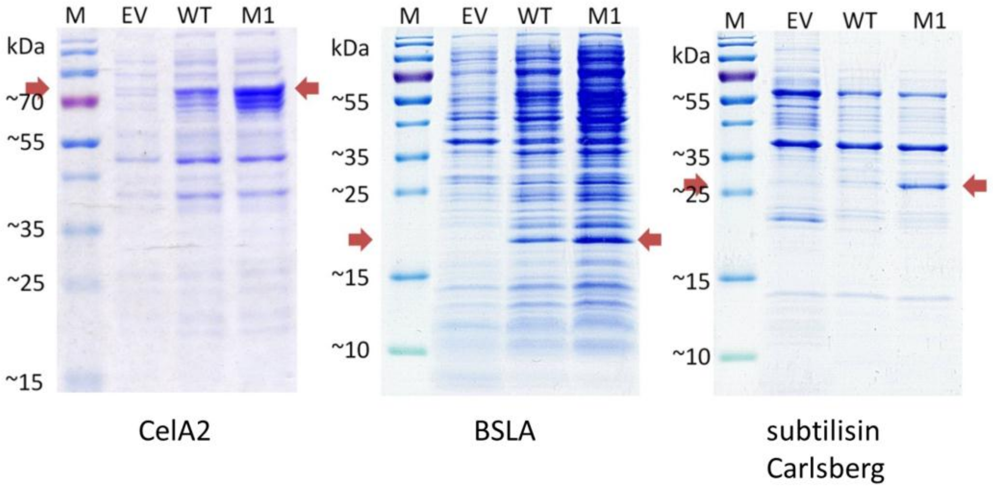

Figure S1: SDS-PAGE of produced cellulase (CelA2), lipase (BSLA) and protease (subtilisin Carlsberg). EV: empty vector control; WT: non-optimized vector constructs; M1: epMEGAWHOP optimized vector constructs. Red arrows indicate the bands of target proteins.

Figure S2: Sequencing results: Alignments of all three vector systems compared to the corresponding 'parents':

pET28a(+)-CelA2 & pET28a(+)M1-CelA2

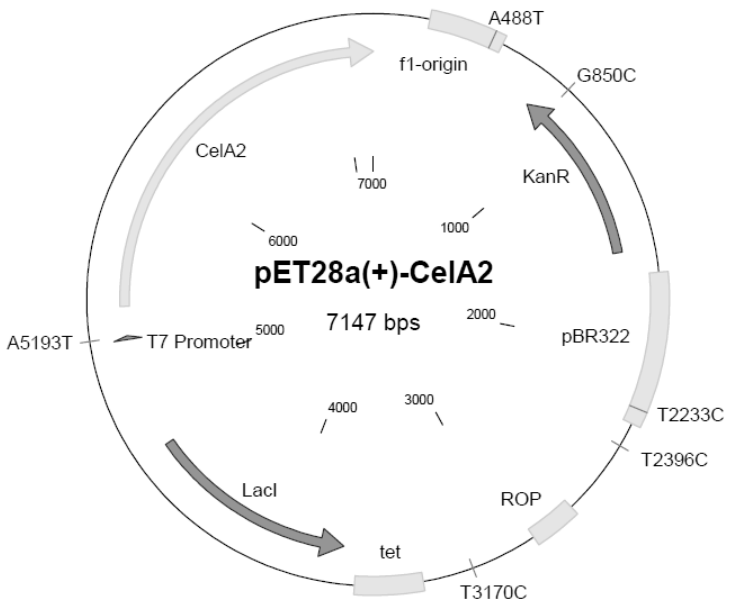

```
pETs28a(+)-CelA2        1 gaattcgagctccgtcgacaagcttgcggccgcactcgagcaccaccaccaccactg
```

```
pET28a(+)-CelA2    1081 ctcgcatcaaccaaaccgttattcattcgtgattgcgcctgagcgagacgaaatacgcga
pET28a(+)-M1       1081 ............................................................

pET28a(+)-CelA2    1141 tcgctgttaaaaggacaattacaaacaggaatcgaatgcaaccggcgcaggaacactgcc
pET28a(+)-M1       1141 ............................................................

pET28a(+)-CelA2    1201 agcgcatcaacaatattttcacctgaatcaggatattcttctaatacctggaatgctgtt
pET28a(+)-M1       1201 ............................................................

pET28a(+)-CelA2    1261 ttcccggggatcgcagtggtgagtaaccatgcatcatcaggagtacggataaaatgcttg
pET28a(+)-M1       1261 ............................................................

pET28a(+)-CelA2    1321 atggtcggaagaggcataaattccgtcagccagtttagtctgaccatctcatctgtaaca
pET28a(+)-M1       1321 ............................................................

pET28a(+)-CelA2    1381 tcattggcaacgctacctttgccatgtttcagaaacaactctggcgcatcgggcttccca
pET28a(+)-M1       1381 ............................................................

pET28a(+)-CelA2    1441 tacaatcgatagattgtcgcacctgattgcccgacattatcgcgagcccatttataccca
pET28a(+)-M1       1441 ............................................................

pET28a(+)-CelA2    1501 tataaatcagcatccatgttggaatttaatcgcggcctagagcaagacgtttcccgttga
pET28a(+)-M1       1501 ............................................................

pET28a(+)-CelA2    1561 atatggctcataacaccccttgtattactgtttatgtaagcagacagttttattgttcat
pET28a(+)-M1       1561 ............................................................

pET28a(+)-CelA2    1621 gaccaaaatcccttaacgtgagttttcgttccactgagcgtcagaccccgtagaaaagat
pET28a(+)-M1       1621 ............................................................

pET28a(+)-CelA2    1681 caaaggatcttcttgagatcctttttttctgcgcgtaatctgctgcttgcaaacaaaaaa
pET28a(+)-M1       1681 ............................................................

pET28a(+)-CelA2    1741 accaccgctaccagcggtggtttgtttgccggatcaagagctaccaactctttttccgaa
pET28a(+)-M1       1741 ............................................................

pET28a(+)-CelA2    1801 ggtaactggcttcagcagagcgcagataccaaatactgtccttctagtgtagccgtagtt
pET28a(+)-M1       1801 ............................................................

pET28a(+)-CelA2    1861 aggccaccacttcaagaactctgtagcaccgcctacatacctcgctctgctaatcctgtt
pET28a(+)-M1       1861 ............................................................

pET28a(+)-CelA2    1921 accagtggctgctgccagtggcgataagtcgtgtcttaccgggttggactcaagacgata
pET28a(+)-M1       1921 ............................................................

pET28a(+)-CelA2    1981 gttaccggataaggcgcagcggtcgggctgaacggggggttcgtgcacacagcccagctt
pET28a(+)-M1       1981 ............................................................

pET28a(+)-CelA2    2041 ggagcgaacgacctacaccgaactgagatacctacagcgtgagctatgagaaagcgccac
pET28a(+)-M1       2041 ............................................................

pET28a(+)-CelA2    2101 gcttcccgaagggagaaaggcggacaggtatccggtaagcggcagggtcggaacaggaga
pET28a(+)-M1       2101 ............................................................

pET28a(+)-CelA2    2161 gcgcacgagggagcttccagggggaaacgcctggtatctttatagtcctgtcgggtttcg
pET28a(+)-M1       2161 ............................................................

pET28a(+)-CelA2    2221 ccacctctgacttgagcgtcgatttttgtgatgctcgtcaggggggcggagcctatggaa
pET28a(+)-M1       2221 ............c...............................................

pET28a(+)-CelA2    2281 aaacgccagcaacgcggcctttttacggttcctggccttttgctggccttttgctcacat
pET28a(+)-M1       2281 ............................................................

pET28a(+)-CelA2    2341 gttctttcctgcgttatcccctgattctgtggataaccgtattaccgcctttgagtgagc
pET28a(+)-M1       2341 .......................................................c....

pET28a(+)-CelA2    2401 tgataccgctcgccgcagccgaacgaccgagcgcagcgagtcagtgagcgaggaagcgga
pET28a(+)-M1       2401 ............................................................
```

```
pET28a(+)-CelA2    2461 agagcgcctgatgcggtattttctccttacgcatctgtgcggtatttcacaccgcaatgg
pET28a(+)-M1       2461 ............................................................

pET28a(+)-CelA2    2521 tgcactctcagtacaatctgctctgatgccgcatagttaagccagtatacactccgctat
pET28a(+)-M1       2521 ............................................................

pET28a(+)-CelA2    2581 cgctacgtgactgggtcatggctgcgccccgacacccgccaacacccgctgacgcgccct
pET28a(+)-M1       2581 ............................................................

pET28a(+)-CelA2    2641 gacgggcttgtctgctcccggcatccgcttacagacaagctgtgaccgtctccgggagct
pET28a(+)-M1       2641 ............................................................

pET28a(+)-CelA2    2701 gcatgtgtcagaggttttcaccgtcatcaccgaaacgcgcgaggcagctgcggtaaagct
pET28a(+)-M1       2701 ............................................................

pET28a(+)-CelA2    2761 catcagcgtggtcgtgaagcgattcacagatgtctgcctgttcatccgcgtccagctcgt
pET28a(+)-M1       2761 ............................................................

pET28a(+)-CelA2    2821 tgagtttctccagaagcgttaatgtctggcttctgataaagcgggccatgttaagggcgg
pET28a(+)-M1       2821 ............................................................

pET28a(+)-CelA2    2881 ttttttcctgtttggtcactgatgcctccgtgtaagggggatttctgttcatgggggtaa
pET28a(+)-M1       2881 ............................................................

pET28a(+)-CelA2    2941 tgataccgatgaaacgagagaggatgctcacgatacgggttactgatgatgaacatgccc
pET28a(+)-M1       2941 ............................................................

pET28a(+)-CelA2    3001 ggttactggaacgttgtgagggtaaacaactggcggtatggatgcggcgggaccagagaa
pET28a(+)-M1       3001 ............................................................

pET28a(+)-CelA2    3061 aaatcactcagggtcaatgccagcgcttcgttaatacagatgtaggtgttccacagggta
pET28a(+)-M1       3061 ............................................................

pET28a(+)-CelA2    3121 gccagcagcatcctgcgatgcagatccggaacataatggtgcagggcgctgacttccgcg
pET28a(+)-M1       3121 .................................................c..........

pET28a(+)-CelA2    3181 tttccagactttacgaaacacggaaaccgaagaccattcatgttgttgctcaggtcgcag
pET28a(+)-M1       3181 ............................................................

pET28a(+)-CelA2    3241 acgttttgcagcagcagtcgcttcacgttcgctcgcgtatcggtgattcattctgctaac
pET28a(+)-M1       3241 ............................................................

pET28a(+)-CelA2    3301 cagtaaggcaaccccgccagcctagccgggtcctcaacgacaggagcacgatcatgcgca
pET28a(+)-M1       3301 ............................................................

pET28a(+)-CelA2    3361 cccgtggggccgccatgccggcgataatggcctgcttctcgccgaaacgtttggtggcgg
pET28a(+)-M1       3361 ............................................................

pET28a(+)-CelA2    3421 gaccagtgacgaaggcttgagcgagggcgtgcaagattccgaataccgcaagcgacaggc
pET28a(+)-M1       3421 ............................................................

pET28a(+)-CelA2    3481 cgatcatcgtcgcgctccagcgaaagcggtcctcgccgaaaatgacccagagcgctgccg
pET28a(+)-M1       3481 ............................................................

pET28a(+)-CelA2    3541 gcacctgtcctacgagttgcatgataaagaagacagtcataagtgcggcgacgatagtca
pET28a(+)-M1       3541 ............................................................

pET28a(+)-CelA2    3601 tgccccgcgcccaccggaaggagctgactgggttgaaggctctcaagggcatcggtcgag
pET28a(+)-M1       3601 ............................................................

pET28a(+)-CelA2    3661 atcccggtgcctaatgagtgagctaacttacattaattgcgttgcgctcactgcccgctt
pET28a(+)-M1       3661 ............................................................

pET28a(+)-CelA2    3721 tccagtcgggaaacctgtcgtgccagctgcattaatgaatcggccaacgcgcggggagag
pET28a(+)-M1       3721 ............................................................

pET28a(+)-CelA2    3781 gcggtttgcgtattgggcgccagggtggtttttcttttcaccagtgagacgggcaacagc
pET28a(+)-M1       3781 ............................................................
```

```
pEТ28a(+)-CelA2    3841 tgattgcccttcaccgcctggccctgagagagttgcagcaagcggtccacgctggtttgc
pET28a(+)-M1       3841 ............................................................

pET28a(+)-CelA2    3901 cccagcaggcgaaaatcctgtttgatggtggttaacggcgggatataacatgagctgtct
pET28a(+)-M1       3901 ............................................................

pET28a(+)-CelA2    3961 tcggtatcgtcgtatcccactaccgagatatccgcaccaacgcgcagcccggactcggta
pET28a(+)-M1       3961 ............................................................

pET28a(+)-CelA2    4021 atggcgcgcattgcgcccagcgccatctgatcgttggcaaccagcatcgcagtgggaacg
pET28a(+)-M1       4021 ............................................................

pET28a(+)-CelA2    4081 atgccctcattcagcatttgcatggtttgttgaaaaccggacatggcactccagtcgcct
pET28a(+)-M1       4081 ............................................................

pET28a(+)-CelA2    4141 tcccgttccgctatcggctgaatttgattgcgagtgagatatttatgccagccagccaga
pET28a(+)-M1       4141 ............................................................

pET28a(+)-CelA2    4201 cgcagacgcgccgagacagaacttaatgggcccgctaacagcgcgatttgctggtgaccc
pET28a(+)-M1       4201 ............................................................

pET28a(+)-CelA2    4261 aatgcgaccagatgctccacgcccagtcgcgtaccgtcttcatgggagaaaataatactg
pET28a(+)-M1       4261 ............................................................

pET28a(+)-CelA2    4321 ttgatgggtgtctggtcagagacatcaagaaataacgccggaacattagtgcaggcagct
pET28a(+)-M1       4321 ............................................................

pET28a(+)-CelA2    4381 tccacagcaatggcatcctggtcatccagcggatagttaatgatcagcccactgacgcgt
pET28a(+)-M1       4381 ............................................................

pET28a(+)-CelA2    4441 tgcgcgagaagattgtgcaccgccgctttacaggcttcgacgccgcttcgttctaccatc
pET28a(+)-M1       4441 ............................................................

pET28a(+)-CelA2    4501 gacaccaccacgctggcacccagttgatcggcgcgagatttaatcgccgcgacaatttgc
pET28a(+)-M1       4501 ............................................................

pET28a(+)-CelA2    4561 gacggcgcgtgcagggccagactggaggtggcaacgccaatcagcaacgactgtttgccc
pET28a(+)-M1       4561 ............................................................

pET28a(+)-CelA2    4621 gccagttgttgtgccacgcggttgggaatgtaattcagctccgccatcgccgcttccact
pET28a(+)-M1       4621 ............................................................

pET28a(+)-CelA2    4681 ttttcccgcgttttcgcagaaacgtggctggcctggttcaccacgcgggaaacggtctga
pET28a(+)-M1       4681 ............................................................

pET28a(+)-CelA2    4741 taagagacaccggcatactctgcgacatcgtataacgttactggtttcacattcaccacc
pET28a(+)-M1       4741 ............................................................

pET28a(+)-CelA2    4801 ctgaattgactctcttccgggcgctatcatgccataccgcgaaaggttttgcgccattcg
pET28a(+)-M1       4801 ............................................................

pET28a(+)-CelA2    4861 atggtgtccgggatctcgacgctctcccttatgcgactcctgcattaggaagcagcccag
pET28a(+)-M1       4861 ............................................................

pET28a(+)-CelA2    4921 tagtaggttgaggccgttgagcaccgccgccgcaaggaatggtgcatgcaaggagatggc
pET28a(+)-M1       4921 ............................................................

pET28a(+)-CelA2    4981 gcccaacagtcccccggccacggggcctgccaccatacccacgccgaaacaagcgctcat
pET28a(+)-M1       4981 ............................................................

pET28a(+)-CelA2    5041 gagcccgaagtggcgagcccgatcttccccatcggtgatgtcggcgatataggcgccagc
pET28a(+)-M1       5041 ............................................................

pET28a(+)-CelA2    5101 aaccgcacctgtggcgccggtgatgccggccacgatgcgtccggcgtagaggatcgagat
pET28a(+)-M1       5101 ............................................................

pET28a(+)-CelA2    5161 ctcgatcccgcgaaattaatacgactcactatagggg aattgtgagcggataacaattcc
pET28a(+)-M1       5161 ................................t...........................
```

```
pET28a(+)-CelA2    5221 cctctagaaataattttgtttaactttaagaaggagatataccatgggtagcagccatca
pET28a(+)-M1       5221 ............................................................

pET28a(+)-CelA2    5281 ccaccatcatcatagcagcggtgaaaatctgtattttcagggtgctagcatgagcgaaga
pET28a(+)-M1       5281 ............................................................

pET28a(+)-CelA2    5341 atggcagacctatgaaaaagaatttaccatgatgtatgaagatgataccctggcacgctt
pET28a(+)-M1       5341 ............................................................

pET28a(+)-CelA2    5401 ttgttttaatctgggtgttcatggtgaaccgaccgaagttgatgaatattttattttga
pET28a(+)-M1       5401 ............................................................

pET28a(+)-CelA2    5461 tgattttaatctggtgatgaccgaaggcaccctgccggaagaagaaaaagaaaatccgat
pET28a(+)-M1       5461 ............................................................

pET28a(+)-CelA2    5521 tcgcctgaatcagattggttatcgtccggaagataataaagtggcctttgtgatttcaga
pET28a(+)-M1       5521 ............................................................

pET28a(+)-CelA2    5581 tgaacgtacctttcgcctgtataccgaagatgatcagctggtttttaccggtaatctgag
pET28a(+)-M1       5581 ............................................................

pET28a(+)-CelA2    5641 cctgtatagcaaagataaagatggtaatccggtggttgatccgaatagcggtgatattac
pET28a(+)-M1       5641 ............................................................

pET28a(+)-CelA2    5701 ccgtgcagcagattttaccagctttgactataatggcaattattatgtgaaagtgcgcaa
pET28a(+)-M1       5701 ............................................................

pET28a(+)-CelA2    5761 tgatgtgagtccggttttggtattgccgaagatgtttatgatgaagttaccaaagccgt
pET28a(+)-M1       5761 ............................................................

pET28a(+)-CelA2    5821 gctgaaaatgttttattatcagcgtagcggtgaactggaagcagaatatgttggtgaact
pET28a(+)-M1       5821 ............................................................

pET28a(+)-CelA2    5881 gtttgcacgtgaagcaggtcatacccagaaagcaagctattatgacccggatgatccggt
pET28a(+)-M1       5881 ............................................................

pET28a(+)-CelA2    5941 ttatggtgatgttgaaattgatgttcatggtggttggtatgatgccggtgattttggtcg
pET28a(+)-M1       5941 ............................................................

pET28a(+)-CelA2    6001 ttatattacaccggcaagcaaagcagttgcagatctgctgctgaccgcagaacattttcc
pET28a(+)-M1       6001 ............................................................

pET28a(+)-CelA2    6061 gaccacccaggaaattgattttggcggtccggaaaaactgctgggtgaaattcgttatga
pET28a(+)-M1       6061 ............................................................

pET28a(+)-CelA2    6121 actggaatggatgctgaaaatgcagaatccggaaaccggtggtgtttatcataaagttac
pET28a(+)-M1       6121 ............................................................

pET28a(+)-CelA2    6181 cgcaaaagatcatgcaccgatgaccattctgccgcatgaagataaaagtccgctgtatct
pET28a(+)-M1       6181 ............................................................

pET28a(+)-CelA2    6241 gagtccggttagcgcacaggcaacaggtgattttgcagcagttatggcctatgcatatcg
pET28a(+)-M1       6241 ............................................................

pET28a(+)-CelA2    6301 catgtataaagatattgatgaagaatttgccaatcagtgtctggatgcagcaatttttgc
pET28a(+)-M1       6301 ............................................................

pET28a(+)-CelA2    6361 atgggaatggctggaagaaaatccgaccgttaatcgttataccgatccgagctttttac
pET28a(+)-M1       6361 ............................................................

pET28a(+)-CelA2    6421 caccggcacctataatgatgttagcgcaaccgatgaacgttattgggcagcagcagaact
pET28a(+)-M1       6421 ............................................................

pET28a(+)-CelA2    6481 gtataaaaccaccggtgatgaaaaatatctgaaatatatggaagataattatctgccgaa
pET28a(+)-M1       6481 ............................................................

pET28a(+)-CelA2    6541 accgggttttggttgggttgaaatgggtagctatgcacatgttgcaattctgaccaatcc
pET28a(+)-M1       6541 ............................................................
```

```
pET28a(+)-CelA2    6601 gctgattgaagaagataccgaactgtatcagaaaaccaaaaatgcctttattaataatgc
pET28a(+)-M1       6601 ............................................................

pET28a(+)-CelA2    6661 ccagagcattctgaaaatttgggaaaaagatggctataaagtggccctggattattatgt
pET28a(+)-M1       6661 ............................................................

pET28a(+)-CelA2    6721 ttggggcagcaataaagatctggcagatcgtgcaatgatgctgatttttgccgatatgat
pET28a(+)-M1       6721 ............................................................

pET28a(+)-CelA2    6781 tgaaccggatccgcgttataaagaagcagttctggatcagattcattattttctgggtcg
pET28a(+)-M1       6781 ............................................................

pET28a(+)-CelA2    6841 caatgccaatgatatttcttatgttaccgcctttggcgaaaaaagcgcacagaatccgca
pET28a(+)-M1       6841 ............................................................

pET28a(+)-CelA2    6901 tcatcgtccgagcgcagttctggaaaccaccgttccgggtatgctggttggtggtccgaa
pET28a(+)-M1       6901 ............................................................

pET28a(+)-CelA2    6961 tgcagagattatgaatgaaatttgggatccggtgagccagattgttgatgaaaatacacc
pET28a(+)-M1       6961 ............................................................

pET28a(+)-CelA2    7021 tccggcacgtcgctatgcagatgttaccggtagctatgccaccaatgaaatttgcattta
pET28a(+)-M1       7021 ............................................................

pET28a(+)-CelA2    7081 ttggaatagtccgctggcctatgttctgggttatgtttatagcctgaataccgtggccga
pET28a(+)-M1       7081 ............................................................

pET28a(+)-CelA2    7141 ataataa
pET28a(+)-M1       7141 .......
```

pET22b(+)-BSLA & pET22b(+)M1-BSLA

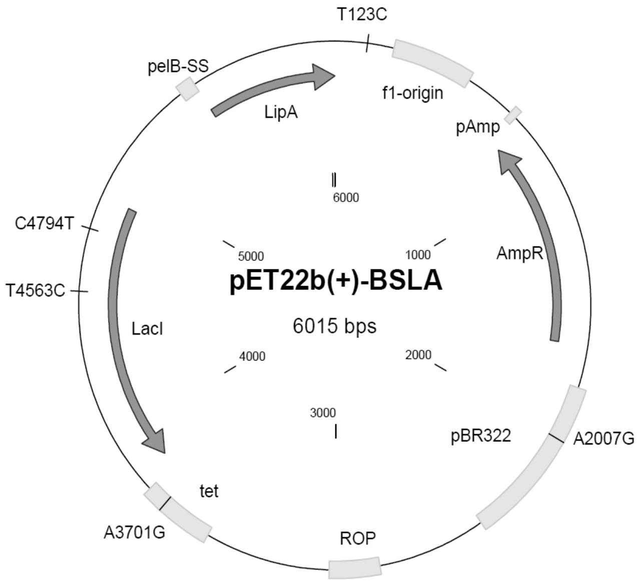

```
pET22b(+)-BSLA      1 tgagagctccgtcgacaagcttgcggccgcactcgagcaccaccaccaccaccactgaga
pet22b(+)-M1        1 ............................................................

pET22b(+)-BSLA     61 tccggctgctaacaaagcccgaaaggaagctgagttggctgctgccaccgctgagcaata
pet22b(+)-M1       61 ............................................................

pET22b(+)-BSLA    121 actagcataaccccttggggcctctaaacgggtcttgaggggttttttgctgaaaggagg
pet22b(+)-M1      121 ..c.........................................................

pET22b(+)-BSLA    181 aactatatccggattggcgaatgggacgcgccctgtagcggcgcattaagcgcggcgggt
pet22b(+)-M1      181 ............................................................

pET22b(+)-BSLA    241 gtggtggttacgcgcagcgtgaccgctacacttgccagcgccctagcgcccgctcctttc
pet22b(+)-M1      241 ............................................................

pET22b(+)-BSLA    301 gctttcttcccttcctttctcgccacgttcgccggctttccccgtcaagctctaaatcgg
pet22b(+)-M1      301 ............................................................

pET22b(+)-BSLA    361 gggctccctttagggttccgatttagtgctttacggcacctcgaccccaaaaaacttgat
pet22b(+)-M1      361 ............................................................

pET22b(+)-BSLA    421 tagggtgatggttcacgtagtgggccatcgccctgatagacggtttttcgccctttgacg
pet22b(+)-M1      421 ............................................................

pET22b(+)-BSLA    481 ttggagtccacgttctttaatagtggactcttgttccaaactggaacaacactcaaccct
pet22b(+)-M1      481 ............................................................

pET22b(+)-BSLA    541 atctcggtctattcttttgatttataagggattttgccgatttcggcctattggttaaaa
pet22b(+)-M1      541 ............................................................

pET22b(+)-BSLA    601 aatgagctgatttaacaaaaatttaacgcgaattttaacaaaatattaacgcttacaatt
pet22b(+)-M1      601 ............................................................

pET22b(+)-BSLA    661 taggtggcacttttcggggaaatgtgcgcggaacccctatttgtttatttttctaaatac
pet22b(+)-M1      661 ............................................................

pET22b(+)-BSLA    721 attcaaatatgtatccgctcatgagacaataaccctgataaatgcttcaataatattgaa
pet22b(+)-M1      721 ............................................................

pET22b(+)-BSLA    781 aaaggaagagtatgagtattcaacatttccgtgtcgcccttattcccttttttgcggcat
pet22b(+)-M1      781 ............................................................

pET22b(+)-BSLA    841 tttgccttcctgtttttgctcacccagaaacgctggtgaaagtaaaagatgctgaagatc
pet22b(+)-M1      841 ............................................................

pET22b(+)-BSLA    901 agttgggtgcacgagtgggttacatcgaactggatctcaacagcggtaagatccttgaga
pet22b(+)-M1      901 ............................................................

pET22b(+)-BSLA    961 gttttcgccccgaagaacgttttccaatgatgagcacttttaaagttctgctatgtggcg
pet22b(+)-M1      961 ............................................................

pET22b(+)-BSLA   1021 cggtattatcccgtattgacgccgggcaagagcaactcggtcgccgcatacactattctc
pet22b(+)-M1     1021 ............................................................
```

```
pET22b(+)-BSLA     1081 agaatgacttggttgagtactcaccagtcacagaaaagcatcttacggatggcatgacag
pet22b(+)-M1       1081 ............................................................

pET22b(+)-BSLA     1141 taagagaattatgcagtgctgccataaccatgagtgataacactgcggccaacttacttc
pet22b(+)-M1       1141 ............................................................

pET22b(+)-BSLA     1201 tgacaacgatcggaggaccgaaggagctaaccgcttttttgcacaacatgggggatcatg
pet22b(+)-M1       1201 ............................................................

pET22b(+)-BSLA     1261 taactcgccttgatcgttgggaaccggagctgaatgaagccataccaaacgacgagcgtg
pet22b(+)-M1       1261 ............................................................

pET22b(+)-BSLA     1321 acaccacgatgcctgcagcaatggcaacaacgttgcgcaaactattaactggcgaactac
pet22b(+)-M1       1321 ............................................................

pET22b(+)-BSLA     1381 ttactctagcttcccggcaacaattaatagactggatggaggcggataaagttgcaggac
pet22b(+)-M1       1381 ............................................................

pET22b(+)-BSLA     1441 cacttctgcgctcggcccttccggctggctggtttattgctgataaatctggagccggtg
pet22b(+)-M1       1441 ............................................................

pET22b(+)-BSLA     1501 agcgtgggtctcgcggtatcattgcagcactggggccagatggtaagccctcccgtatcg
pet22b(+)-M1       1501 ............................................................

pET22b(+)-BSLA     1561 tagttatctacacgacggggagtcaggcaactatggatgaacgaaatagacagatcgctg
pet22b(+)-M1       1561 ............................................................

pET22b(+)-BSLA     1621 agataggtgcctcactgattaagcattggtaactgtcagaccaagtttactcatatatac
pet22b(+)-M1       1621 ............................................................

pET22b(+)-BSLA     1681 tttagattgatttaaaacttcatttttaatttaaaaggatctaggtgaagatcctttttg
pet22b(+)-M1       1681 ............................................................

pET22b(+)-BSLA     1741 ataatctcatgaccaaaatcccttaacgtgagttttcgttccactgagcgtcagaccccg
pet22b(+)-M1       1741 ............................................................

pET22b(+)-BSLA     1801 tagaaaagatcaaaggatcttcttgagatcctttttttctgcgcgtaatctgctgcttgc
pet22b(+)-M1       1801 ............................................................

pET22b(+)-BSLA     1861 aaacaaaaaaaccaccgctaccagcggtggtttgtttgccggatcaagagctaccaactc
pet22b(+)-M1       1861 ............................................................

pET22b(+)-BSLA     1921 tttttccgaaggtaactggcttcagcagagcgcagataccaaatactgtccttctagtgt
pet22b(+)-M1       1921 ............................................................

pET22b(+)-BSLA     1981 agccgtagttaggccaccacttcaagaactctgtagcaccgcctacatacctcgctctgc
pet22b(+)-M1       1981 ..........................g.................................

pET22b(+)-BSLA     2041 taatcctgttaccagtggctgctgccagtggcgataagtcgtgtcttaccgggttggact
pet22b(+)-M1       2041 ............................................................

pET22b(+)-BSLA     2101 caagacgatagttaccggataaggcgcagcggtcgggctgaacggggggttcgtgcacac
pet22b(+)-M1       2101 ............................................................

pET22b(+)-BSLA     2161 agcccagcttggagcgaacgacctacaccgaactgagatacctacagcgtgagctatgag
pet22b(+)-M1       2161 ............................................................

pET22b(+)-BSLA     2221 aaagcgccacgcttcccgaagggagaaaggcggacaggtatccggtaagcggcagggtcg
pet22b(+)-M1       2221 ............................................................

pET22b(+)-BSLA     2281 gaacaggagagcgcacgagggagcttccagggggaaacgcctggtatctttatagtcctg
pet22b(+)-M1       2281 ............................................................

pET22b(+)-BSLA     2341 tcgggtttcgccacctctgacttgagcgtcgatttttgtgatgctcgtcaggggggcgga
pet22b(+)-M1       2341 ............................................................

pET22b(+)-BSLA     2401 gcctatggaaaaacgccagcaacgcggcctttttacggttcctggccttttgctggcctt
pet22b(+)-M1       2401 ............................................................
```

```
pET22b(+)-BSLA     2461 ttgctcacatgttctttcctgcgttatcccctgattctgtggataaccgtattaccgcct
pet22b(+)-M1       2461 ............................................................

pET22b(+)-BSLA     2521 ttgagtgagctgataccgctcgccgcagccgaacgaccgagcgcagcgagtcagtgagcg
pet22b(+)-M1       2521 ............................................................

pET22b(+)-BSLA     2581 aggaagcggaagagcgcctgatgcggtattttctccttacgcatctgtgcggtatttcac
pet22b(+)-M1       2581 ............................................................

pET22b(+)-BSLA     2641 accgcaatggtgcactctcagtacaatctgctctgatgccgcatagttaagccagtatac
pet22b(+)-M1       2641 ............................................................

pET22b(+)-BSLA     2701 actccgctatcgctacgtgactgggtcatggctgcgccccgacacccgccaacacccgct
pet22b(+)-M1       2701 ............................................................

pET22b(+)-BSLA     2761 gacgcgccctgacgggcttgtctgctcccggcatccgcttacagacaagctgtgaccgtc
pet22b(+)-M1       2761 ............................................................

pET22b(+)-BSLA     2821 tccgggagctgcatgtgtcagaggttttcaccgtcatcaccgaaacgcgcgaggcagctg
pet22b(+)-M1       2821 ............................................................

pET22b(+)-BSLA     2881 cggtaaagctcatcagcgtggtcgtgaagcgattcacagatgtctgcctgttcatccgcg
pet22b(+)-M1       2881 ............................................................

pET22b(+)-BSLA     2941 tccagctcgttgagtttctccagaagcgttaatgtctggcttctgataaagcgggccatg
pet22b(+)-M1       2941 ............................................................

pET22b(+)-BSLA     3001 ttaagggcggtttttcctgtttggtcactgatgcctccgtgtaagggggatttctgttc
pet22b(+)-M1       3001 ............................................................

pET22b(+)-BSLA     3061 atgggggtaatgataccgatgaaacgagagaggatgctcacgatacgggttactgatgat
pet22b(+)-M1       3061 ............................................................

pET22b(+)-BSLA     3121 gaacatgcccggttactggaacgttgtgagggtaaacaactggcggtatggatgcggcgg
pet22b(+)-M1       3121 ............................................................

pET22b(+)-BSLA     3181 gaccagagaaaaatcactcagggtcaatgccagcgcttcgttaatacagatgtaggtgtt
pet22b(+)-M1       3181 ............................................................

pET22b(+)-BSLA     3241 ccacagggtagccagcagcatcctgcgatgcagatccggaacataatggtgcagggcgct
pet22b(+)-M1       3241 ............................................................

pET22b(+)-BSLA     3301 gacttccgcgtttccagactttacgaaacacggaaaccgaagaccattcatgttgttgct
pet22b(+)-M1       3301 ............................................................

pET22b(+)-BSLA     3361 caggtcgcagacgttttgcagcagcagtcgcttcacgttcgctcgcgtatcggtgattca
pet22b(+)-M1       3361 ............................................................

pET22b(+)-BSLA     3421 ttctgctaaccagtaaggcaaccccgccagcctagccgggtcctcaacgacaggagcacg
pet22b(+)-M1       3421 ............................................................

pET22b(+)-BSLA     3481 atcatgcgcacccgtggggccgccatgccggcgataatggcctgcttctcgccgaaacgt
pet22b(+)-M1       3481 ............................................................

pET22b(+)-BSLA     3541 ttggtggcgggaccagtgacgaaggcttgagcgagggcgtgcaagattccgaataccgca
pet22b(+)-M1       3541 ............................................................

pET22b(+)-BSLA     3601 agcgacaggccgatcatcgtcgcgctccagcgaaagcggtcctcgccgaaaatgacccag
pet22b(+)-M1       3601 ............................................................

pET22b(+)-BSLA     3661 agcgctgccggcacctgtcctacgagttgcatgataaagaagacagtcataagtgcggcg
pet22b(+)-M1       3661 ........................................g...................

pET22b(+)-BSLA     3721 acgatagtcatgccccgcgcccaccggaaggagctgactgggttgaaggctctcaagggc
pet22b(+)-M1       3721 ............................................................

pET22b(+)-BSLA     3781 atcggtcgagatcccggtgcctaatgagtgagctaacttacattaattgcgttgcgctca
pet22b(+)-M1       3781 ............................................................
```

```
pET22b(+)-BSLA     3841 ctgcccgctttccagtcgggaaacctgtcgtgccagctgcattaatgaatcggccaacgc
pet22b(+)-M1       3841 ............................................................

pET22b(+)-BSLA     3901 gcggggagaggcggtttgcgtattgggcgccagggtggtttttcttttcaccagtgagac
pet22b(+)-M1       3901 ............................................................

pET22b(+)-BSLA     3961 gggcaacagctgattgcccttcaccgcctggccctgagagagttgcagcaagcggtccac
pet22b(+)-M1       3961 ............................................................

pET22b(+)-BSLA     4021 gctggtttgccccagcaggcgaaaatcctgtttgatggtggttaacggcgggatataaca
pet22b(+)-M1       4021 ............................................................

pET22b(+)-BSLA     4081 tgagctgtcttcggtatcgtcgtatcccactaccgagatatccgcaccaacgcgcagccc
pet22b(+)-M1       4081 ............................................................

pET22b(+)-BSLA     4141 ggactcggtaatggcgcgcattgcgcccagcgccatctgatcgttggcaaccagcatcgc
pet22b(+)-M1       4141 ............................................................

pET22b(+)-BSLA     4201 agtgggaacgatgccctcattcagcatttgcatggtttgttgaaaaccggacatggcact
pet22b(+)-M1       4201 ............................................................

pET22b(+)-BSLA     4261 ccagtcgccttcccgttccgctatcggctgaatttgattgcgagtgagatatttatgcca
pet22b(+)-M1       4261 ............................................................

pET22b(+)-BSLA     4321 gccagccagacgcagacgcgccgagacagaacttaatgggcccgctaacagcgcgatttg
pet22b(+)-M1       4321 ............................................................

pET22b(+)-BSLA     4381 ctggtgacccaatgcgaccagatgctccacgcccagtcgcgtaccgtcttcatgggagaa
pet22b(+)-M1       4381 ............................................................

pET22b(+)-BSLA     4441 aataatactgttgatgggtgtctggtcagagacatcaagaaataacgccggaacattagt
pet22b(+)-M1       4441 ............................................................

pET22b(+)-BSLA     4501 gcaggcagcttccacagcaatggcatcctggtcatccagcggatagttaatgatcagccc
pet22b(+)-M1       4501 ............................................................

pET22b(+)-BSLA     4561 actgacgcgttgcgcgagaagattgtgcaccgccgctttacaggcttcgacgccgcttcg
pet22b(+)-M1       4561 ..c.........................................................

pET22b(+)-BSLA     4621 ttctaccatcgacaccaccacgctggcacccagttgatcggcgcgagatttaatcgccgc
pet22b(+)-M1       4621 ............................................................

pET22b(+)-BSLA     4681 gacaatttgcgacggcgcgtgcagggccagactggaggtggcaacgccaatcagcaacga
pet22b(+)-M1       4681 ............................................................

pET22b(+)-BSLA     4741 ctgtttgcccgccagttgttgtgccacgcggttgggaatgtaattcagctccgccatcgc
pet22b(+)-M1       4741 .....................................................t......

pET22b(+)-BSLA     4801 cgcttccactttttcccgcgttttcgcagaaacgtggctggcctggttcaccacgcggga
pet22b(+)-M1       4801 ............................................................

pET22b(+)-BSLA     4861 aacggtctgataagagacaccggcatactctgcgacatcgtataacgttactggtttcac
pet22b(+)-M1       4861 ............................................................

pET22b(+)-BSLA     4921 attcaccaccctgaattgactctcttccgggcgctatcatgccataccgcgaaaggtttt
pet22b(+)-M1       4921 ............................................................

pET22b(+)-BSLA     4981 gcgccattcgatggtgtccgggatctcgacgctctcccttatgcgactcctgcattagga
pet22b(+)-M1       4981 ............................................................

pET22b(+)-BSLA     5041 agcagcccagtagtaggttgaggccgttgagcaccgccgccgcaaggaatggtgcatgca
pet22b(+)-M1       5041 ............................................................

pET22b(+)-BSLA     5101 aggagatggcgcccaacagtcccccggccacggggcctgccaccatacccacgccgaaac
pet22b(+)-M1       5101 ............................................................

pET22b(+)-BSLA     5161 aagcgctcatgagcccgaagtggcgagcccgatcttccccatcggtgatgtcggcgatat
pet22b(+)-M1       5161 ............................................................
```

```
pET22b(+)-BSLA     5221 aggcgccagcaaccgcacctgtggcgccggtgatgccggccacgatgcgtccggcgtaga
pet22b(+)-M1       5221 ............................................................

pET22b(+)-BSLA     5281 ggatcgagatctcgatcccgcgaaattaatacgactcactataggggaattgtgagcgga
pet22b(+)-M1       5281 ............................................................

pET22b(+)-BSLA     5341 taacaattcccctctagaaataattttgtttaactttaagaaggagatatacatatgaaa
pet22b(+)-M1       5341 ............................................................

pET22b(+)-BSLA     5401 tacctgctgccgaccgctgctgctggtctgctgctcctcgctgcccagccggcgatggcc
pet22b(+)-M1       5401 ............................................................

pET22b(+)-BSLA     5461 atggatatcgctgaacacaatccagtcgttatggttcacggtattggagggcatcattc
pet22b(+)-M1       5461 ............................................................

pET22b(+)-BSLA     5521 aattttgcgggaattaagagctatctcgtatctcagggctggtcgcgggacaagctgtat
pet22b(+)-M1       5521 ............................................................

pET22b(+)-BSLA     5581 gcagttgatttttgggacaagacaggcacaaattataacaatggaccggtattatcacga
pet22b(+)-M1       5581 ............................................................

pET22b(+)-BSLA     5641 tttgtgcaaaaggttttagatgaaacgggtgcgaaaaaagtggatattgtcgctcacagc
pet22b(+)-M1       5641 ............................................................

pET22b(+)-BSLA     5701 atgggggcgcgaacacactttactacataaaaaatctggacggcggaaataaagttgca
pet22b(+)-M1       5701 ............................................................

pET22b(+)-BSLA     5761 aacgtcgtgacgcttggcggcgcgaaccgtttgacgacaggcaaggcgcttccgggaaca
pet22b(+)-M1       5761 ............................................................

pET22b(+)-BSLA     5821 gatccaaatcaaaagattttatacacatccatttacagcagtgccgatatgattgtcatg
pet22b(+)-M1       5821 ............................................................

pET22b(+)-BSLA     5881 aattacttatcaagattagatggtgctagaaacgttcaaatccatggcgttggacacatc
pet22b(+)-M1       5881 ............................................................

pET22b(+)-BSLA     5941 ggccttctgtacagcagccaagtcaacagcctgattaaagaagggctgaacggcgggggc
pet22b(+)-M1       5941 ............................................................

pET22b(+)-BSLA     6001 cagaatacgaattaa
pet22b(+)-M1       6001 ...............
```

pHY-scarlsberg & pHYM1-scarlsberg

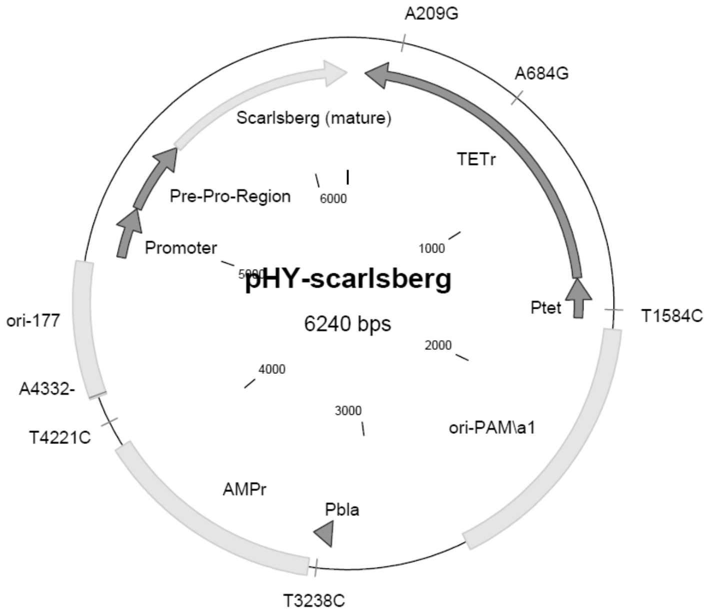

```
pHY-scarlsberg       1 cataccccgggaattcctgttataaaaaaaggatcaattttgaactctctcccaaagttg
pHY M1-scarlsber     1 ............................................................

pHY-scarlsberg      61 atcccttaacgatttagaaatccctttgagaatgtttatatacattcaaggtaaccagcc
pHY M1-scarlsber    61 ............................................................

pHY-scarlsberg     121 aactaatgacaatgattcctgaaaaaagtaataacaaattactatacagataagttgact
pHY M1-scarlsber   121 ............................................................

pHY-scarlsberg     181 gatcaacttccataggtaacaacctttgatcaagtaagggtatggataataaaccaccta
pHY M1-scarlsber   181 ............................g...............................

pHY-scarlsberg     241 caattgcaatacctgttccctctgataaaaagctggtaaagttaagcaaactcattccag
pHY M1-scarlsber   241 ............................................................

pHY-scarlsberg     301 caccagcttcctgctgtttcaagctacttgaaacaattgttgatataactgttttggtga
pHY M1-scarlsber   301 ............................................................

pHY-scarlsberg     361 acgaaagcccacctaaaacaaatacgattataattgtcatgaaccatgatgttgtttcta
pHY M1-scarlsber   361 ............................................................

pHY-scarlsberg     421 aaagaaaggaagcagttaaaaagctaacagaaagaaatgtaactccgatgtttaacacgt
pHY M1-scarlsber   421 ............................................................

pHY-scarlsberg     481 ataaaggacctcttctatcaacaagtatcccaccaatgtagccgaaaataatgacactca
pHY M1-scarlsber   481 ............................................................

pHY-scarlsberg     541 ttgttccagggaaaataattacacttccgatttcggcagtacttagctggtgaacatctt
pHY M1-scarlsber   541 ............................................................

pHY-scarlsberg     601 tcatcatataaggaaccatagagacaaaccctgctactgttccaaatataattcccccac
pHY M1-scarlsber   601 ............................................................

pHY-scarlsberg     661 aaagaactccaatcataaaaggtatattttccctaatccgggatcaacaaaaggatctg
pHY M1-scarlsber   661 .......................g....................................

pHY-scarlsberg     721 ttactttcctgatatgttttacaaatatcaggaatgacagcacgctaacgataagaaaag
pHY M1-scarlsber   721 ............................................................

pHY-scarlsberg     781 aaatgctatatgatgttgtaaacaacataaaaaatacaatgcctacagacattagtataa
pHY M1-scarlsber   781 ............................................................

pHY-scarlsberg     841 ttcctttgatatcaaaatgaccttttatccttacttctttctttaataatttcataagaa
pHY M1-scarlsber   841 ............................................................

pHY-scarlsberg     901 acggaacagtgataattgttatcataggaatgagtagaagataggaccaatgaatataat
pHY M1-scarlsber   901 ............................................................

pHY-scarlsberg     961 gggctatcattccaccaatcgctggaccgactccttctcccatggctactatcgatccaa
pHY M1-scarlsber   961 ............................................................

pHY-scarlsberg    1021 taagaccaaatgctttacccctatttcctttggaatatagcgcgcaactacaaccatta
pHY M1-scarlsber  1021 ............................................................
```

```
pHY-scarlsberg     1081 cgagtgctggaaatgcagctgcaccagccccttgaataaaacgagccataataagtaagg
pHY M1-scarlsber   1081 ............................................................

pHY-scarlsberg     1141 aaaagaaagaatggccaacaaacccaattaccgacccgaaacaatttattataattccaa
pHY M1-scarlsber   1141 ............................................................

pHY-scarlsberg     1201 ataggagtaaccttttgatgcctaattgatcagatagctttccatatacagctgttccaa
pHY M1-scarlsber   1201 ............................................................

pHY-scarlsberg     1261 tggaaaaggttaacataaaggctgtgttcacccagtttgtactcgcaggtggtttattaa
pHY M1-scarlsber   1261 ............................................................

pHY-scarlsberg     1321 aatcatttgcaatatcaggtaatgagacgttcaaaaccatttcatttaatacgctaaaaa
pHY M1-scarlsber   1321 ............................................................

pHY-scarlsberg     1381 aagataaaatgcaaagccaaattaaaatttggttgtgtcgtaaattcgattgtgaatagg
pHY M1-scarlsber   1381 ............................................................

pHY-scarlsberg     1441 atgtattcacatttcaccctccaataatgagggcagacgtagtttatagggttaatgata
pHY M1-scarlsber   1441 ............................................................

pHY-scarlsberg     1501 cgcttccctcttttaattgaaccctgttacattcattattcattacacttcataattaat
pHY M1-scarlsber   1501 ............................................................

pHY-scarlsberg     1561 tcctcctaaacttgattaaacatttaccacatataaactaagttttaaattcagtatt
pHY M1-scarlsber   1561 ......................c.....................................

pHY-scarlsberg     1621 tcatcacttatacaacaatatggcccgtttgttgaactactctttaataaaataattttt
pHY M1-scarlsber   1621 ............................................................

pHY-scarlsberg     1681 ccgttcccaattccacattgcaataatagaaaatccatcttcatcggctttttcgtcatc
pHY M1-scarlsber   1681 ............................................................

pHY-scarlsberg     1741 atctgtatgaatcaaatcgccttcttctgtgtcatcaaggtttaatttttatgtatttc
pHY M1-scarlsber   1741 ............................................................

pHY-scarlsberg     1801 ttttaacaaaccaccataggagattaaccttttacggtgtaaaccttcctccaaatcaga
pHY M1-scarlsber   1801 ............................................................

pHY-scarlsberg     1861 caaacgtttcaaattcttttcttcatcatcggtcataaaatccgtatcctttacaggata
pHY M1-scarlsber   1861 ............................................................

pHY-scarlsberg     1921 ttttgcagtttcgtcaattgccgattgtatatccgatttatatttatttttcggtcgaat
pHY M1-scarlsber   1921 ............................................................

pHY-scarlsberg     1981 catttgaacttttacatttggatcatagtctaatttcattgcctttttccaaaattgaat
pHY M1-scarlsber   1981 ............................................................

pHY-scarlsberg     2041 ccattgtttttgattcacgtagttttctgtattcttaaaataagttggttccacacatac
pHY M1-scarlsber   2041 ............................................................

pHY-scarlsberg     2101 caatacatgcatgtgctgattataagaattatctttattatttattgtcacttccgttgc
pHY M1-scarlsber   2101 ............................................................

pHY-scarlsberg     2161 acgcataaaaccaacaagattttattaatttttttatattgcatcattcggcgaaatcc
pHY M1-scarlsber   2161 ............................................................

pHY-scarlsberg     2221 ttgagccatatctgacaaactcttatttaattcttcgccatcataaacattttaactgt
pHY M1-scarlsber   2221 ............................................................

pHY-scarlsberg     2281 taatgtgagaaacaaccaacgaactgttggcttttgtttaataacttcagcaacaacctt
pHY M1-scarlsber   2281 ............................................................

pHY-scarlsberg     2341 ttgtgactgaatgccatgtttcattgctctcctccagttgcacattggacaaagcctgga
pHY M1-scarlsber   2341 ............................................................

pHY-scarlsberg     2401 tttacaaaaccacactcgatacaactttctttcgcctgtttcacgattttgtttatactc
pHY M1-scarlsber   2401 ............................................................
```

```
pHY-scarlsberg      2461 taatatttcagcacaatcttttactctttcagcctttttaaattcaagaatatgcagaag
pHY M1-scarlsber    2461 ............................................................

pHY-scarlsberg      2521 ttcaaagtaatcaacattagcgattttcttttctctccatggtctcacttttccactttt
pHY M1-scarlsber    2521 ............................................................

pHY-scarlsberg      2581 tgtcttgtccactaaaacccttgatttttcatctgaataaatgctactattaggacacat
pHY M1-scarlsber    2581 ............................................................

pHY-scarlsberg      2641 aatattaaaagaaacccccatctatttagttatttgtttggtcacttataactttaacag
pHY M1-scarlsber    2641 ............................................................

pHY-scarlsberg      2701 atggggtttttctgtgcaaccaattttaagggttttcaatactttaaaacacatacatac
pHY M1-scarlsber    2701 ............................................................

pHY-scarlsberg      2761 caacacttcaacgcacctttcagcaactaaaataaaaatgacgttatttctatatgtatc
pHY M1-scarlsber    2761 ............................................................

pHY-scarlsberg      2821 aagataagaaagaacaagttcaaaaccatcaaaaaaagacaccttttcaggtgctttttt
pHY M1-scarlsber    2821 ............................................................

pHY-scarlsberg      2881 tattttataaactcattccctgatctcgacttcgttctttttttacctctcggttatgag
pHY M1-scarlsber    2881 ............................................................

pHY-scarlsberg      2941 ttagttcaaattcgttctttttaggttctaaatcgtgtttttcttggaattgtgctgttt
pHY M1-scarlsber    2941 ............................................................

pHY-scarlsberg      3001 tatcctttaccttgtctacaaaccccttaaaaacgtttttaaaggcttttaagccgtctg
pHY M1-scarlsber    3001 ............................................................

pHY-scarlsberg      3061 tacgttccttaaggaattattccttagtgctttctaggttaatgtcatgataataatggt
pHY M1-scarlsber    3061 ............................................................

pHY-scarlsberg      3121 ttcttagacgtcaggtggcacttttcggggaaatgtgcgcggaacccctatttgtttatt
pHY M1-scarlsber    3121 ............................................................

pHY-scarlsberg      3181 tttctaaatacattcaaatatgtatccgctcatgagacaataaccctgataaatgcttca
pHY M1-scarlsber    3181 .........................................................c..

pHY-scarlsberg      3241 ataatattgaaaaaggaagagtatgagtattcaacatttccgtgtcgcccttattccctt
pHY M1-scarlsber    3241 ............................................................

pHY-scarlsberg      3301 ttttgcggcattttgccttcctgtttttgctcacccagaaacgctggtgaaagtaaaaga
pHY M1-scarlsber    3301 ............................................................

pHY-scarlsberg      3361 tgctgaagatcagttgggtgcacgagtgggttacatcgaactggatctcaacagcggtaa
pHY M1-scarlsber    3361 ............................................................

pHY-scarlsberg      3421 gatccttgagagttttcgccccgaagaacgttttccaatgatgagcacttttaaagttct
pHY M1-scarlsber    3421 ............................................................

pHY-scarlsberg      3481 gctatgtggcgcggtattatcccgtgttgacgccgggcaagagcaactcggtcgccgcat
pHY M1-scarlsber    3481 ............................................................

pHY-scarlsberg      3541 acactattctcagaatgacttggttgagtactcaccagtcacagaaaagcatcttacgga
pHY M1-scarlsber    3541 ............................................................

pHY-scarlsberg      3601 tggcatgacagtaagagaattatgcagtgctgccataaccatgagtgataacactgcggc
pHY M1-scarlsber    3601 ............................................................

pHY-scarlsberg      3661 caacttacttctgacaacgatcggaggaccgaaggagctaaccgcttttttgcacaacat
pHY M1-scarlsber    3661 ............................................................

pHY-scarlsberg      3721 ggggatcatgtaactcgccttgatcgttgggaaccggagctgaatgaagccataccaaa
pHY M1-scarlsber    3721 ............................................................

pHY-scarlsberg      3781 cgacgagcgtgacaccacgatgcctgcagcaatggcaacaacgttgcgcaaactattaac
pHY M1-scarlsber    3781 ............................................................
```

```
pHY-scarlsberg     3841 tggcgaactacttactctagcttcccggcaacaattaatagactggatggaggcggataa
pHY M1-scarlsber   3841 ............................................................

pHY-scarlsberg     3901 agttgcaggaccacttctgcgctcggcccttccggctggctggtttattgctgataaatc
pHY M1-scarlsber   3901 ............................................................

pHY-scarlsberg     3961 tggagccggtgagcgtgggtctcgcggtatcattgcagcactggggccagatggtaagcc
pHY M1-scarlsber   3961 ............................................................

pHY-scarlsberg     4021 ctcccgtatcgtagttatctacacgacggggagtcaggcaactatggatgaacgaaatag
pHY M1-scarlsber   4021 ............................................................

pHY-scarlsberg     4081 acagatcgctgagataggtgcctcactgattaagcattggtaactgtcagaccaagttta
pHY M1-scarlsber   4081 ............................................................

pHY-scarlsberg     4141 ctcatatatactttagattgatttaaaacttcatttttaatttaaaaggatctaggtgaa
pHY M1-scarlsber   4141 ............................................................

pHY-scarlsberg     4201 gatcctttttgataatctcatgaccaaaatcccttaacgtgagttttcgttccactgagc
pHY M1-scarlsber   4201 ....................c.......................................

pHY-scarlsberg     4261 gtcagaccccttaataagatgatcttcttgagatcgttttggtctgcgcgtaatctcttg
pHY M1-scarlsber   4261 ............................................................

pHY-scarlsberg     4321 ctctgaaaacgaaaaaaccgccttgcagggaggtttttcgaaggttctctgagctaccaa
pHY M1-scarlsber   4321 ...........-................................................

pHY-scarlsberg     4381 ctctttgaaccgaggtaactggcttggaggagcgcagtcaccaaaacttgtcctttcagt
pHY M1-scarlsber   4380 ............................................................

pHY-scarlsberg     4441 ttagccttaaccggcgcatgacttcaagactaactcctctaaatcaattaccagtggctg
pHY M1-scarlsber   4440 ............................................................

pHY-scarlsberg     4501 ctgccagtggtgcttttgcatgtctttccgggttggactcaagacgatagttaccggata
pHY M1-scarlsber   4500 ............................................................

pHY-scarlsberg     4561 aggcgcagcggtcggactgaacggggggttcgtgcatacagtccagcttggagcgaactg
pHY M1-scarlsber   4560 ............................................................

pHY-scarlsberg     4621 cctacccggaactgagtgtcaggcgtggaatgagacaaacgcggccataacagcggaatg
pHY M1-scarlsber   4620 ............................................................

pHY-scarlsberg     4681 acaccggtaaaccgaaaggcaggaacaggagagcgcacgagggagccgccaggggggaaac
pHY M1-scarlsber   4680 ............................................................

pHY-scarlsberg     4741 gcctggtatctttatagtcctgtcgggtttcgccaccactgatttgagcgtcagatttcg
pHY M1-scarlsber   4740 ............................................................

pHY-scarlsberg     4801 tgatgcttgtcaggggggcggagcctatggaaaaacggctttgcccaagcttctagagat
pHY M1-scarlsber   4800 ............................................................

pHY-scarlsberg     4861 ctgcaggtcgacggatccgacctctttccctgccaggctgaagcggtctattcatacttt
pHY M1-scarlsber   4860 ............................................................

pHY-scarlsberg     4921 cgaactgaacattttctaaaacagttattaataaccaaaaaatttaaattggtcctcc
pHY M1-scarlsber   4920 ............................................................

pHY-scarlsberg     4981 aaaaaaataggcctaccatataattcatttttttctataataaattaacagaataattg
pHY M1-scarlsber   4980 ............................................................

pHY-scarlsberg     5041 gaatagattatattatccttctatttaaattattctgaataaagaggaggagagtgagta
pHY M1-scarlsber   5040 ............................................................

pHY-scarlsberg     5101 atgatgaggaaaaagagtttttggcttgggatgctgacggccttcatgctcgtgttcacg
pHY M1-scarlsber   5100 ............................................................

pHY-scarlsberg     5161 atggcattcagcgattccgcttctgctgctcaaccggcgaaaaatgttgaaaaggattat
pHY M1-scarlsber   5160 ............................................................
```

```
pHY-scarlsberg     5221 attgtcggatttaagtcaggagtgaaaaccgcatctgtcaaaaaggacatcatcaaagag
pHY M1-scarlsber   5220 ............................................................

pHY-scarlsberg     5281 agcggcggaaaagtggacaagcagtttagaatcatcaacgcggcaaaagcgaagctagac
pHY M1-scarlsber   5280 ............................................................

pHY-scarlsberg     5341 aaagaagcgcttaaggaagtcaaaaatgatccggatgtcgcttatgtggaagaggatcat
pHY M1-scarlsber   5340 ............................................................

pHY-scarlsberg     5401 gtggcccatgcgctagcgcaaaccgttccttacggcattcctctcattaaagcggacaaa
pHY M1-scarlsber   5400 ............................................................

pHY-scarlsberg     5461 gtgcaggctcaaggctttaagggagcgaatgtaaaagtagccgtcctggatacaggaatc
pHY M1-scarlsber   5460 ............................................................

pHY-scarlsberg     5521 caagcttctcatccggacttgaacgtagtcggcggagcaagctttgtggctggcgaagct
pHY M1-scarlsber   5520 ............................................................

pHY-scarlsberg     5581 tataacaccgacggcaacggacacggcacacatgttgccggtacagtagctgcgcttgac
pHY M1-scarlsber   5580 ............................................................

pHY-scarlsberg     5641 aatacaacgggtgtattaggcgttgcgccaagcgtatccttgtacgcggttaaagtactg
pHY M1-scarlsber   5640 ............................................................

pHY-scarlsberg     5701 aattcaagcggaagcggatcatacagcggcattgtaagcggaatcgagtgggcgacaaca
pHY M1-scarlsber   5700 ............................................................

pHY-scarlsberg     5761 aacggcatggatgttatcaatatgagccttggggggagcatcaggctcgacagcgatgaaa
pHY M1-scarlsber   5760 ............................................................

pHY-scarlsberg     5821 caggcagtcgacaatgcatatgcaagaggggttgtcgttgtagctgcagcagggaacagc
pHY M1-scarlsber   5820 ............................................................

pHY-scarlsberg     5881 ggatcttcaggaaacacgaatacaattggctatcctgcgaaatacgattctgtcatcgct
pHY M1-scarlsber   5880 ............................................................

pHY-scarlsberg     5941 gttggtgcggtagactctaacagcaacagagcttcattttccagcgtcggagcagagctt
pHY M1-scarlsber   5940 ............................................................

pHY-scarlsberg     6001 gaagtcatggctcctggcgcaggcgtatacagcacttacccaacgaacacttatgcaaca
pHY M1-scarlsber   6000 ............................................................

pHY-scarlsberg     6061 ttgaacggaacgtcaatggcttctcctcatgtagcgggagcagcagctttgatcttgtca
pHY M1-scarlsber   6060 ............................................................

pHY-scarlsberg     6121 aaacatccgaacctttcagcttcacaagtccgcaaccgtctctccagcacggcgacttat
pHY M1-scarlsber   6120 ............................................................

pHY-scarlsberg     6181 ttgggaagctccttctactatgggaaaggtctgatcaatgtcgaagctgccgctcaataa
pHY M1-scarlsber   6180 ............................................................
```
